# Dispersant and Protective Roles of Amphiphilic Poly(ethylene phosphate) Block Copolymers in Polyester/Bone Mineral Composites

**DOI:** 10.3390/ijms241311175

**Published:** 2023-07-06

**Authors:** Ilya Nifant’ev, Alexander Tavtorkin, Pavel Komarov, Egor Kretov, Sofia Korchagina, Maria Chinova, Dmitry Gavrilov, Pavel Ivchenko

**Affiliations:** 1 A.V. Topchiev Institute of Petrochemical Synthesis RAS, 29 Leninsky Pr., 119991 Moscow, Russia; tavtorkin@yandex.ru (A.T.); komarrikov@yandex.ru (P.K.); egor.kretov.02@mail.ru (E.K.); korchagina@ips.ac.ru (S.K.); chinova@yandex.ru (M.C.); gavrosdm@gmail.com (D.G.); phpasha1@yandex.ru (P.I.); 2 Chemistry Department, M.V. Lomonosov Moscow State University, 1–3 Leninskie Gory, 119991 Moscow, Russia; 3 Faculty of Chemistry, National Research University Higher School of Economics, Myasnitskaya St. 20, 101100 Moscow, Russia

**Keywords:** carbonated apatite, composites, mechanical characteristics, thermal degradation, polylactide, poly(ε-caprolactone), polyphosphodiesters, ring-opening polymerization

## Abstract

Composites of synthetic bone mineral substitutes (BMS) and biodegradable polyesters are of particular interest for bone surgery and orthopedics. Manufacturing of composite scaffolds commonly uses mixing of the BMS with polymer melts. Melt processing requires a high homogeneity of the mixing, and is complicated by BMS-promoted thermal degradation of polymers. In our work, poly(*L*-lactide) (PLLA) and poly(ε-caprolactone) (PCL) composites reinforced by commercial β-tricalcium phosphate (βTCP) or synthesized carbonated hydroxyapatite with hexagonal and plate-like crystallite shapes (hCAp and pCAp, respectively) were fabricated using injection molding. pCAp-based composites showed advanced mechanical and thermal characteristics, and the best set of mechanical characteristics was observed for the PLLA-based composite containing 25 wt% of pCAp. To achieve compatibility of polyesters and pCAp, reactive block copolymers of PLLA or PCL with poly(*tert-*butyl ethylene phosphate) (C1 and C2, respectively) were introduced to the composite. The formation of a polyester-*b*-poly(ethylene phosphoric acid) (PEPA) compatibilizer during composite preparation, followed by chemical binding of PEPA with pCAp, have been proved experimentally. The presence of 5 wt% of the compatibilizer provided deeper homogenization of the composite, resulting in a marked increase in strength and moduli as well as a more pronounced nucleation effect during isothermal crystallization. The use of C1 increased the thermal stability of the PLLA-based composite, containing 25 wt% of pCAp. In view of positive impacts of polyester-*b*-PEPA on composite homogeneity, mechanical characteristics, and thermal stability, polyester-*b*-PEPA will find application in the further development of composite materials for bone surgery and orthopedics.

## 1. Introduction

Human cortical bone is a complex entity comprising the bone mineral (BM), organic components, and water [1,2]. The very complex structure of the bone represents triple-helix tropocollagen molecules, self-assembled into collagen fibrils (50–100 nm in diameter and up to several μm in length), and non-collagenous proteins; collagen serves as a scaffold for BM deposition in the form of nano-sized plate-like crystallites [3,4]. Phosphorylated protein osteopontin serves as a reactive biopolymer–compatibilizer for BM and collagen [4].

A major focus of bone surgery is to restore the functionality of the bone in terms of its optimal strength parameters and anatomical shape. Bone healing is a complex morphogenetic process [5], and its success, above all, is determined by the placement and size of bone injury [6]. Treatment of critical-sized bone defects often requires the use of scaffolds, tailored to the morphology and size of bone damage, and/or surgical construction elements. These articles must be biocompatible and might be biodegradable or not; in the latter case, the second surgical intervention for implant removal may be needed [2,7]. Similarity of the mechanical properties of bone substitutes and bone tissue is also desirable [8,9,10], e.g., conventional metal articles have the mismatch of an elastic modulus of metal with the surrounding bone, which may cause bone destruction and implant failure [11].

The “gold standard” treatment using autogeneous bone grafts is confined by the relative scarcity of sources, post-operative side effects, and a prolonged grafting time [12]. An ideal synthetic bone substitute should be biocompatible, biodegradable, and mechanically stable, supporting biological activities such as cell attachment, migration, and growth; osteoconductivity is a necessary property for bone substitute scaffolds [6,8,13]. It is quite natural that osteoconductive synthetic minerals, containing Ca^2+^, PO_4_^3−^, and other “biophilic” ions, are seen as prospective bone mineral substitutes (BMS) [14,15]. These BMS can be combined with other inorganic phases, i.e., ZrO_2_ [16], SiC [17], Al_2_O_3_ [18], metallic [19] and carbon [14] fibers, bioglasses [20], and natural and synthetic polymers [15,21]. At first glance, when natural or synthetic biodegradable polymers and BMS are used in composite formulations, the materials obtained mimic the bone tissue [14,15,22,23,24,25]. However, the formation of natural bone tissues, perfected by millions of years of evolution, is actually superior to simple laboratory solutions, and metal-based composites are still actual weight-bearing bone substitutes [26,27].

**Figure 1:**
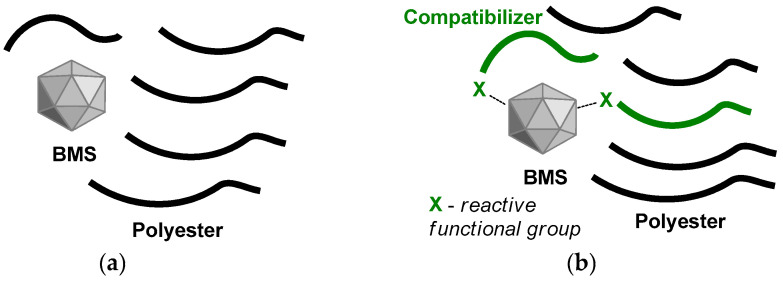
Polyester/BMS composites without (**a**) and with the use of a compatibilizer (**b**).

Preparation of comparatively strong polyester/BMS scaffolds is based on two methods, injection molding with the use of the desired shape of the casting and 3D additive manufacturing [28]. Both techniques are based on melt processing and require highly homogenized source materials. However, it is inevitable for BMS to agglomerate in a polymer matrix because of poor phase compatibility between BMS and polyesters. In addition, at high temperatures of the composite molding, synthetic BMS can promote degradation of polyesters [29,30,31,32,33]. The composite characteristics might be improved with the use of compatibilizers with surface modification for BMS that provide better coupling between polyester and BMS (Figure 1). All components of such systems deserve separate attention.

Polyester components, e.g., the non-toxic biodegradable synthetic polymers poly(*L*-lactide) (PLLA) [21,23,24,25,34], poly(*L*-lactide-*co*-glycolide) (PLGA) [35], and poly(ε-caprolactone) (PCL) [21,23,25], are attractive materials for the production of implants. However, PLLA and PLGA have several drawbacks, such as a low cell affinity, a poor osteoconductivity, and suboptimal mechanical properties for load-bearing applications [7,36]. Furthermore, a release of acidic byproducts during biodegradation of polyesters (especially PLGA) may cause an inflammatory response in surrounding tissues [37].

Among BMS, FDA-approved [38] hydroxyapatite (HAp) was studied in many works [15,25,32,39,40]. However, pure “stochiometric” HAp of the formula Ca_10_(PO_4_)_6_(OH)_2_ is not quite appropriate [41] because of its high degree of crystallinity, which entails a reduced solubility/biodegradability [1], poor mechanical properties [14], and even cytotoxicity in specific cases [42]. Consequently, other inorganic phases, for instance, β-tricalcium phosphate (βTCP) [31,43], octacalcium phosphate [44], et al., have been studied as BMS. Among synthetic BMS, carbonated apatite (CAp) is perfectly suited for use in composites because biomimetic cortical bone apatite is actually a CAp [1,14,45,46]. CAp is also attractive due to its basic characteristic, the potential ability to neutralize acidic products of the degradation of polyesters. In 2017, CAp was approved as an artificial bone substitute by the Pharmaceuticals and Medical Devices Agency of Japan [1]. However, polyester/CAp composites are virtually unexplored because of the synthetic unavailability of perfectly shaped nano-/microsized CAp materials until recently [47].

In recent years, to improve compatibility of the polyesters and inorganic fillers, the treatment of BMS with organic acids [48,49] including PLA oligomers [50,51,52], dodecanol [53], polydopamine [54,55], alkyl alkoxy silanes [56], isocyanates [57], carboxymethyl-β-cyclodextrin [58], and poly(ethylene glycol)-*b*-PLLA [59], as well as the initiation of the ring-opening polymerization (ROP) of *L*-lactide [60,61,62,63,64,65,66] or ε-caprolactone [67] by nano-sized calcium phosphates, were proposed. The strong binding of the polymer to the surface of BMS can be achieved by compatibilizers containing nature-like acidic –P(O)(OH)_2_ or >P(O)(OH) fragments. Similar compatibilizers have been widely used in dentistry [68,69]; however, until recently, amphiphilic derivatives of phosphoric acid have not been studied as compatibilizers for BMS/polyester composites. Among phosphorus-containing polyacids, poly(phosphodiester)s seem to be promising in view of their BM affinity [69,70,71,72,73,74,75] and osteoinductivity [69,75,76,77]. In a recent study [78], we demonstrated a positive impact of an amphiphilic copolymer containing PCL and poly(ethylene phosphoric acid) (PEPA) blocks on the morphology of PCL/HAp fibrous scaffolds, prepared with the electrospinning technique.

In the present work, we tried to find ways of further improving polyester/BMS composites. To achieve the goals of this study, we synthesized carbonated apatites of a different morphology (hexagonal and plate-like, hCAp and pCAp, respectively), prepared polyester/BMS composites using injection molding with the use of β-TCP (as a commercially available standard, see Figure S6 in Supplementary Materials) and hCAp and pCAp as BMS, and studied the influence of the BMS type and polyester-*b*-PEPA compatibilizer on mechanical, thermal, and rheological characteristics of the composites. To solve these problems, we propose the surface modification of the BMS with the use of PEPA-based compatibilizers.

## 2. Results and Discussion

### 2.1. Preparation of the Composites

#### 2.1.1. Synthesis of CAp

The preparation of CAp samples was based on our recent work devoted to the hydrothermal synthesis of micro-sized CAp species with diverse morphologies [47] with the use of CaCO_3_ as a Ca^2+^ and CO_3_^2−^ source, Na_2_[EDTA]·2H_2_O for calcite dissolution and for the control of crystallite formation, NaH_2_PO_4_ as a PO_4_^3−^ source, and Na_2_CO_3_ or NaHCO_3_ as a CO_3_^2−^ source and pH support, according to general Equation (1).

(10 − x) Ca^2+^ + x Na^+^ + (6 − x) PO_4_^3−^ + x CO_3_^2−^ + 2 OH^−^ → Ca_10−x_Na_x_(PO_4_)_6−x_(CO_3_)_x_(OH)_2_ (1)

**Figure 2:**
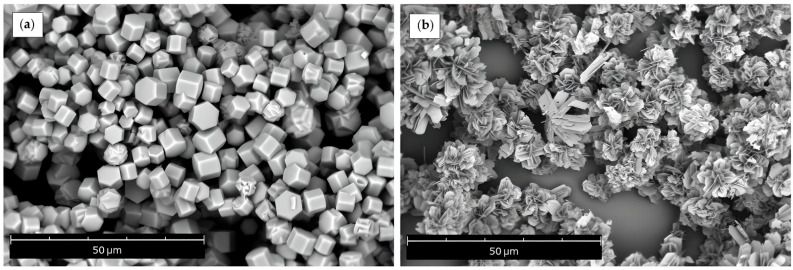
SEM images of (**a**) hexagonal and (**b**) plate-like carbonated apatite (hCAp and pCAp, respectively).

However, when trying to scale up the method proposed previously [47], we had the problem of a low reproducibility of crystallite sizes and morphologies even at minimal changes in starting conditions and component ratios. In the present study, we developed an efficient method of the synthesis of CAp that provides obtaining two types of CAp in the amount of tens of grams. Hexagonal prismatic CAp (hCAp, Figure 2a and Figure S7 in Supplementary Materials) with an aspect ratio of 0.8–1 and a width of 4–8 μm was steadily obtained with the use of Na_2_CO_3_ (starting pH ~9) in two stages: pre-treatment at 100 °C within 1 h with subsequent filtration and a hydrothermal reaction at 150 °C within 5 h. At the initial stage of the process (after 0.5 h), formation of the microspherical precipitate was detected (Figure S8 in Supplementary Materials). Aggregates of plate-like crystallites (pCAp, Figure 2b and Figure S9 in Supplementary Materials) were synthesized with the use of NaHCO_3_ (starting pH ~6) at 140 °C within 24 h, and filtration of the reaction solution before hydrothermal treatment was essential for reproducibility of the crystallite size and morphology. The typical yields of hCAp and pCAp were 19.6 ± 0.3 and 18.6 ± 0.4%, respectively. X-ray diffraction studies confirmed a B-type CAp identity (Figures S10 and S11 in Supplementary Materials), and TGA and FT-IR data (Figure S12 in Supplementary Materials) showed that incorporation of CO_3_^2−^ anions was higher for hCAp in comparison with pCAp (Table 1). As shown previously, increased CO_3_^2−^ content in CAp facilitates its resorption and replacement by the natural bone tissue [79,80], so that at first sight, hCAp seemed to be preferable for biomedical applications. On the other hand, pCAp crystallites contained a mainly Ca^2+^-enriched surface with an enhanced ability of the bonding with polyesters and PEPA [81].

#### 2.1.2. Synthesis of Polyester-b-poly(^t^BuOEP) Compatibilizer

**Scheme 1:**
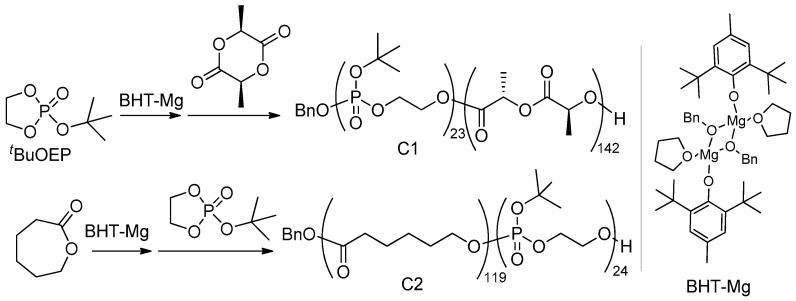
Synthesis of amphiphilic block copolymers C1 and C2.

The synthesis of polyester-*b*-poly(*^t^*BuOEP) copolymers with the use of the ROP catalyst BHT-Mg (Scheme 1) was based on our previous work [82]. Starting the synthesis of compatibilizers, we thought that a sufficient degree of polymerization (*DP*_n_) for the polyester fragment will be at least 100 monomer units, whereas for the poly(*^t^*BuOEP) fragment, *DP*_n_ ~20 is sufficient to provide further binding with BMS. Due to a significant difference in reaction mechanisms and reactivities of *L*-lactide (*L*-LA), ε-caprolactone (εCL), and *^t^*BuOEP in BHT-Mg-catalyzed ROP [83,84], *^t^*BuOEP was introduced as a first and second comonomer in the syntheses of block copolymers with *L*-LA and εCL, respectively (Scheme 1). As a result, copolymers BnO(*^t^*BuOEP)_23_-*b*-(*L*-LA)_142_ (C1) and BnO(εCL)_119_-*b*-(*^t^*BuOEP)_24_ (C2) were synthesized, notably having a narrow molecular weight distribution due to the use of modified methods of *^t^*BuOEP purification (Figure S1 in Supplementary Materials) and copolymerization (see Section 3.3 and Section S1 in Supplementary Materials).

#### 2.1.3. Preparation and Molding of the Composites

PLLA-based composite samples PL01–PL14 (Table 2) were prepared with homogenization of the BMS suspension in a THF solution of PLLA; when using C1 as a compatibilizer, suspensions were stirred for 1 additional hour at 90 °C to provide complete elimination of isobutylene with a formation of PEPA. After elimination of THF at 100 °C, the residues were further homogenized (see Section 3.5) and transferred into an injection molding system. Standard Type V dumbbell-shaped (ASTM D638-14) and 60 × 10 × 1 mm rectangular plate samples were prepared for tensile and flexural tests, respectively. Eight-specimen series were prepared for each composite formulation.

### 2.2. Characteristics of the PLLA-Based Composites

#### 2.2.1. Mechanical Properties of PLLA/BMS Composites

The mechanical characteristics of PLLA and dense PLLA/BMS composites, reported in scientific periodicals, essentially depend on the methods of sample preparation and mechanical testing. So, for instance, PLLA reported values of the tensile strength σ_t_ (48–154 MPa), tensile modulus *E*_t_ (0.6–3.8 GPa), flexural strength σ_f_ (64–258 MPa), and flexural modulus *E*_f_ (3.2–6.5 GPa) range widely [22,32,34,40,85,86,87,88]. To compare mechanical properties of commercial PLLA and its composites with βTCP, hCAp, and pCAp, we prepared and tested eight samples for each formulation.

**Figure 3:**
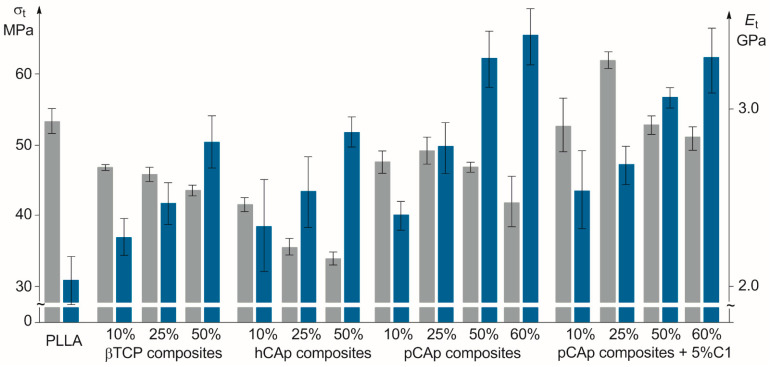
Dependences of the tensile strength (grey) and modulus (blue) on wt% of βTCP, hCAp, and pCAp in PLLA-based composites. Average squared deviations are presented as bounded segments.

Initially, three series of PLLA-based samples containing 10, 25, and 50 wt% of BMS (PL01–PL09, Table 2) were studied. For βTCP and hCAp, we observed a decrease in σ_t_ with an increase in BMS content (Figure 3), similarly to PLLA composites with HAp [50], but for pCAp, the value of σ_t_ passed through the local maximum when the content of the filler was 25 wt%; a similar pattern was observed previously for PLLA composites with surface-modified HAp [50,87]. The values of σ_t_ were lower for hCAp-based samples PL04–PL06 probably due to their content of relatively large mineral particles with a low surface area. PL01–PL03 and PL07–PL09 series had close σ_t_ values with a slight advantage of pCAp-based composites, most likely because of proximity of the sizes of βTCP and pCAp particles. The values of the tensile modulus *E*_t_ of PLLA/BMS composites increased in comparison with PLLA, with almost linear correlations between *E*_t_ and wt% of BMS for all types of filler. pCAp-based PL09 with 50 wt% pCAp content had a maximum *E*_t_ value of 3.29 GPa in the series. An increase in pCAp content to 60 wt% (sample PL10, Table 2) resulted in a further decrease in σ_t_ and increase in *E*_t_, but accuracy of the measurements was significantly lower (the samples with 60 wt% content of βTCP and hCAp were also prepared, but their mechanical tests failed because of irreproducibility of the data obtained).

Usually, homogeneous materials with a lower σ_t_ demonstrate higher values of elongation at break ε [89]. However, this pattern is not fully applicable to composites, including PLLA/BMS systems. So, for example, the addition of low (<10 wt%) amounts of HAp resulted in an increase in ε values in comparison with PLLA [48]. In the present study, for composites containing 10 wt% of BMS, ε values were slightly higher than the ε value of PLLA, and decreased with a further increase in BMS content; a similar pattern was observed previously [87].

**Figure 4:**
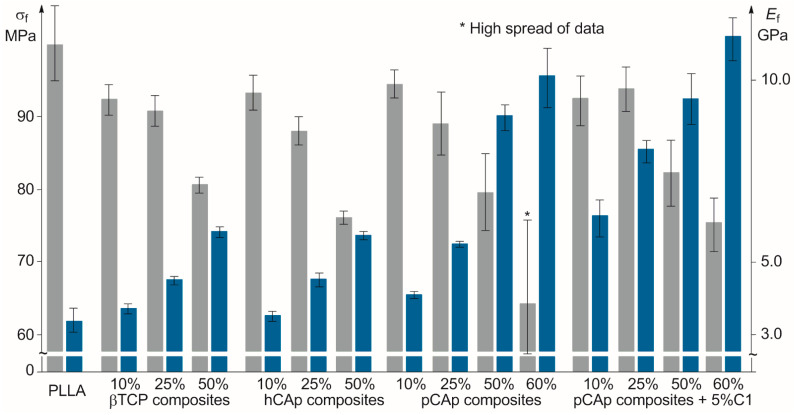
Dependences of the flexural strength (grey) and modulus (blue) on wt% of βTCP, hCAp, and pCAp in PLLA-based composites. Average squared deviations are presented as bounded segments.

In earlier studies, a decrease in σ_f_ (or little effect) with increasing BMS content in PLLA/BMS composites [32,85,90], as well as a positive impact of BMS content on the *E*_f_ value [11,32,85,86], have been demonstrated. In our flexural tests, σ_f_ of the samples PL01–PL09 decreased with an increase in BMS content (Figure 4). This effect was most pronounced for hCAp-containing samples. On the other hand, pCAp had a stronger impact on the flexural modulus; a threefold increase in the *E*_f_ value relative to PLLA (10.1 vs. 3.3 GPa) was detected for PL10 containing 60 wt% of pCAp. We assumed that a substantial loss of strength, observed for PL07–PL10, can be attributed to the specific morphology of pCAp crystallites that represent clusters of plates (Figure 2). Apparently, these clusters do not break down at dispersion and molding stages, and it is their destruction during a bending test that leads to cracks of the PLLA matrix and weakens the composite.

#### 2.2.2. Mechanical Properties of PLLA/C1/pCAp Composites

Based on mechanical test results, we considered pCAp as the most prospective filler among the three BMS under study, if we are able to solve the problem of flexural strength reducing. The addition of the amphiphilic block copolymer C1 to composite formulation was seen as a possible way to improve mechanical characteristics of pCAp-based materials. We proposed that initially lipophilic polyester-*b*-poly(*^t^*BuOEP) copolymers should decompose with a formation of amphiphilic block copolymers of PEPA during composite preparation. The in situ-obtained compatibilizer should bind tightly to the BMS surface, along with that the polyester blocks remain unchanged and provide compatibility with the polyester matrix.

We prepared four samples with 10, 25, 50, and 60 wt% of pCAp, containing 5 wt% of C1 (samples PL11–PL14), and made tensile and bending tests (Table 2, Figure 3 and Figure 4). Tensile tests showed that the addition of C1 results in a substantial increase in σ_t_ relative to PL07–PL10; the values of *E*_t_ for PL11–PL14 were slightly lower in comparison with PL07–PL10. Composite PL12 with 25 wt% content of pCAp had the highest σ_t_ (62.2 MPa) that exceeded σ_t_ of PLLA. Tensile strengths of PL13 (50 wt% of pCAp) and PLLA were close, but *E*_t_ of PL13 was one and a half times higher. Based on the bending test results, one can conclude that the use of compatibilizer C1 results in an increase in both σ_f_ and *E*_f_ in PL12 and PL13 in comparison with PL08 and PL09. The effect of C1 can be explained by chemical bonding of the PEPA block in the macromolecule, formed from C1 during composite preparation, to the pCAp surface, and by combining of the PLLA block of C1 with the PLLA matrix. We propose that these interactions should result in destruction of plate aggregates and more uniform distribution of pCAp crystallites in the composite.

The surfaces of the samples PL09 and PL13 were observed with SEM. As can be seen in Figures S14 and S15 in Supplementary Materials, the inorganic phase seems well-dispersed even at 50 wt% content of pCAp in both samples. The measurements of the contact angles of PLLA and composites with 50 wt% of the filler (see Table S1 in Supplementary Materials) confirm coating of pCAp particles by PLLA; the lowest contact angle (64.2°) was detected for PL13. It is clear that these observations do not prove PEPA–CAp chemical bonding in C1-based composites.

#### 2.2.3. Evidence for PEPA–CAp Bonding in PLLA/C1/pCAp Composites

**Figure 5:**
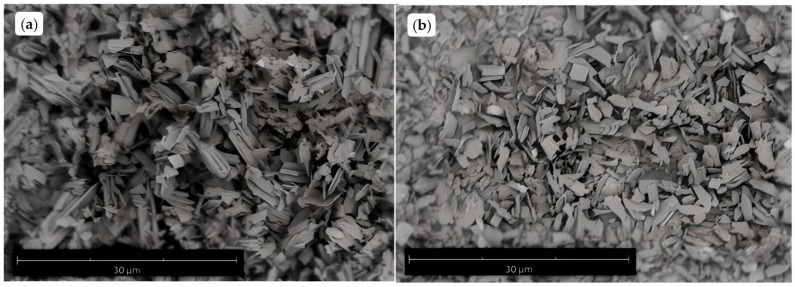
SEM images of the mineral residues after treatment of the samples PL09 (**a**) and PL13 (**b**) with THF.

The marked difference between composites PL09 and PL13, prepared with and without C1, became visible when analyzing SEM images of the fracture surfaces, obtained after a bending test (Figure S16 in Supplementary Materials), that confirm the activation of particle debonding followed by plastic void growth around the fracture plane for PL09; similar phenomena have been observed previously for PLLA/HAp composites [88]. A significant difference of filler morphology and distribution in PL09 and PL13 was also observed after complete elimination of PLLA by dissolution in THF. As can be seen in Figure 5a, aggregation of pCAp crystallites in the absence of C1 was retained during composite preparation, whereas in the presence of C1, pCAp splices turned out to be completely broken (Figure 5b). In this way, C1 provided deeper homogenization of the pCAp, serving as a surfactant. Note that a similar pattern was observed previously when using PLA-grafted HAp nanoparticles in composite formulations [50].

**Figure 6:**
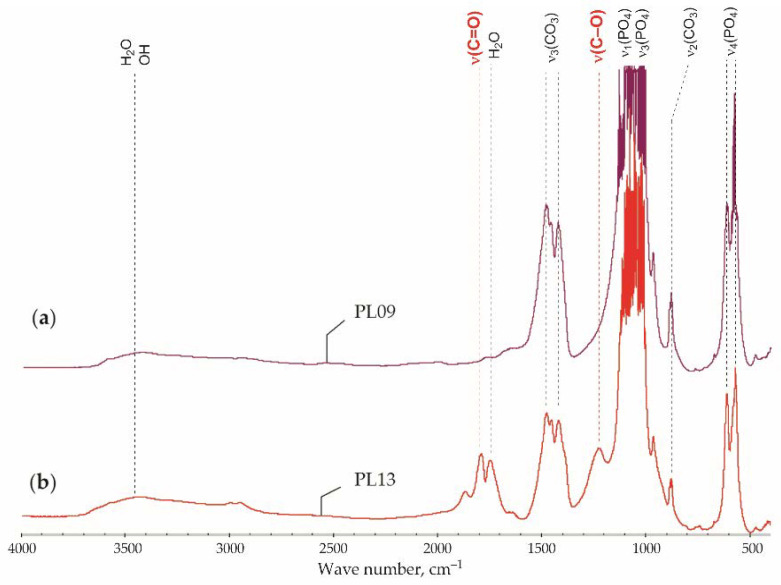
FT-IR spectra of the solid residues after extraction of PL09 (**a**) and PL13 (**b**) with a CH_2_Cl_2_/toluene mixture.

Experiments on the extraction of polymers with the use of a non-solvating CH_2_Cl_2_/toluene mixture confirmed chemical binding between BMS and PLLA-*b*-PEPA. FT-IR spectra of the mineral residues after extraction of PL09 and PL13 (Figure 6) were significantly different, and the latter included characteristic signals of PLLA at ~1800 (>C=O) and 1220 (C–O) cm^−1^ [91] due to the presence of PEPA-*b*-PLLA bound to pCAp. The evaporated extract of PL13 did not contain phosphorus (^31^P NMR data), thus confirming degradation of C1 with a formation of a PEPA block during preparation and molding of the composite.

#### 2.2.4. Thermal Properties of PLLA-Based Composites

As mechanical properties can be attributed not only to the introduction of the rigid filler but also to an increase in polymer crystallinity, which is determined by the nature of BMS and their content, we studied the thermal properties of PLLA-based composites. One of the eight samples, prepared and tested for each composite formulation, was selected according to the criterion of proximity of its mechanical characteristics to the average values.

For all composite samples, we observed a slight lowering of the glass transition temperature *T*_g_ (Table 3) that was attributed previously [48] to softening of the polymer chains in the presence of the filler. The degree of crystallinity χ_c_ was calculated from the difference between Δ*H*_m_ and Δ*H*_c_ values for the sample and Δ*H*_m_ of 100% crystalline PLLA (93 J·g^−1^, [32]). For pCAp-based composites PL07–PL09 and PL11–PL13, we observed correlation between σ_t_ and χ_c_ with a maximum for PL08 and PL12 containing 25 wt% of pCAp. The presence of the PEPA-*b*-PLLA compatibilizer (Table 3, samples PL11–PL13) did not have a significant effect on the thermal properties of the composites. Other composites containing 25% BMS (PL02 and PL05) demonstrated a lower nucleating effect.

**Figure 7:**
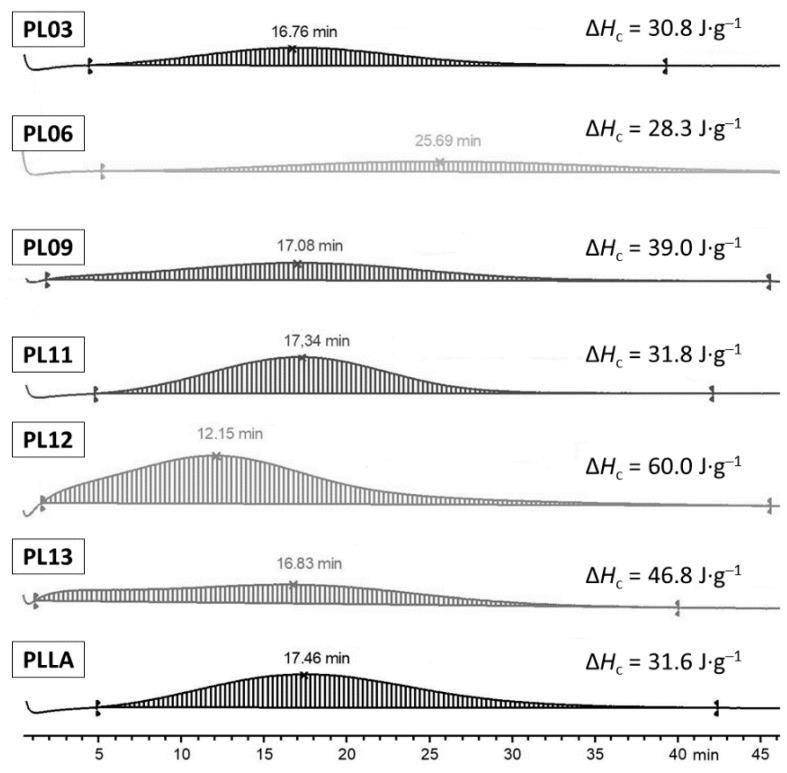
DSC plots of isothermal crystallization at 110 °C for PLLA and PLLA-based composites.

As shown in a number of works, the presence of well-dispersed BMS facilitates crystallization of the polyester, thus demonstrating a nucleation effect of BMS [32,48,92]. By the results of DSC measurements, the cold crystallization of the composites was observed at lower temperatures *T*_c_ in comparison with *T*_c_ of PLLA, and the cold crystallization enthalpy Δ*H*_c_ values were higher than Δ*H*_c_ of PLLA; in this way, BMS particles served as crystallization promoters. Additional experiments on isothermal crystallization were conducted for PLLA and PL09 at 100, 110, and 120 °C (see Figure S17 in Supplementary Materials). At 120 °C, for PL09, we observed a mild nucleation effect with a crystallization peak at 32 min (χ_c_ = 19%). At 110 °C, the difference was the most visible (for PLLA and PL09, χ_c_ comprised 33 and 43%, and crystallization peaks were detected at 17.5 and 17.1 min, respectively). At 100 °C, a nucleation effect of pCAp was particularly pronounced, maximum crystallization peaks for PLLA and PL09 were detected at 14 and 11.1 min, and χ_c_ values were 30 and 56%, respectively. Since the quality of 3D printing is essentially determined by the behavior of the melt at the beginning of the formation of the solid phase, we made a comparative study of PLLA and PLLA-based composites at the temperature of 110 °C under which the difference becomes visible (Figure 7). The temperature of 120 °C, used previously in comparative studies of PLLA and PLLA/BMS composites [48,92], seemed less suitable.

As can be seen in Figure 7, at 110 °C, β-TCP being added in an equal amount had almost no effect on crystallization of the composite PL03 and hCAp (PL06) slowed down crystallization, but pCAp (PL09) provided slightly earlier crystallization with a higher χ_c_. The use of the C1 compatibilizer resulted in a marked increase in the χ_c_ values (PL11–PL13), and for 25 wt% BMS formulation (PL12), the maximum of the crystallization peak was detected 5 min earlier in comparison with PLLA, and the χ_c_ value was 65%. Note that the sample PL12 with 25 wt% pCAp content had a higher σ_t_ value in comparison with PL11 and PL13. It is also worth noting that the immediate onset of crystallization at 110 °C was observed only for pCAp-based samples PL12 and PL13, which allows for considering pCAp as a prospective filler for additive manufacturing of biomedical scaffolds, in combination with an appropriate compatibilizer.

#### 2.2.5. Rheology of PLLA-Based Composites

**Figure 8:**
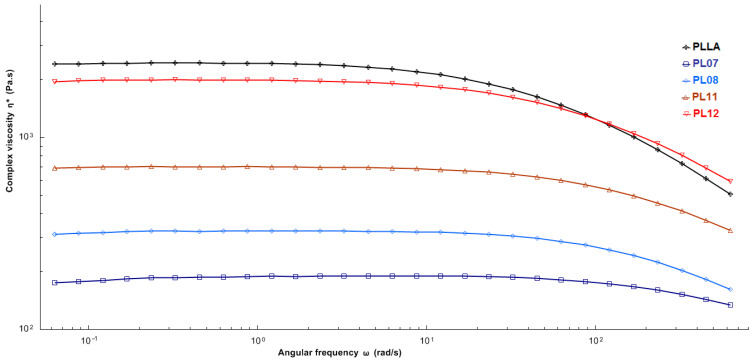
Log (η*) vs. log (ω) for PLLA and PLLA/pCAp composites.

In this study, the rheological properties of neat PLLA and composites of PLLA with pCAp (PL07, PL08, PL11, and PL12) were assessed with viscosity in the function of the shear rate. Figure 8 shows the relationship between the complex viscosity (η*) with the frequency ω (rad·s^−1^) for all samples; the values of viscosity were not sensible to the increase in the shear rate from 0.06 s^−1^ to 10 s, and at higher shear rates, non-Newtonian behavior arose (see also the dependences of the viscosity and shear stress from the shear rate in Figures S18 and S19 in Supplementary Materials). All composite samples had lower viscosities in comparison with neat PLLA, and similar behavior was detected previously for HAp/PLLA composites with 5–25% BMS loading [33]. However, we observed the effect of the use of C1 in composite formulations that is shown in higher viscosity values when compared with composites prepared by mixing PLLA and CAp without the C1 compatibilizer.

**Figure 9:**
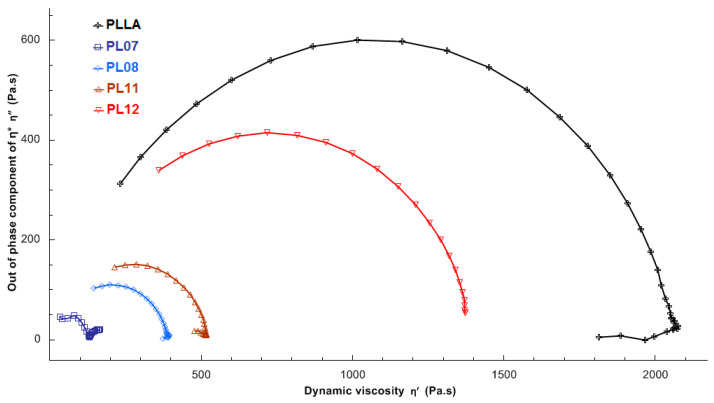
Cole–Cole representations of the dynamic viscosity of PLA and its composites.

For qualitative evaluation of the dispersion of pCAp in the PLLA matrix, we plotted a Cole–Cole diagram (Figure 9). This analysis allows detecting the presence of particle agglomerates in polymeric systems [88]. In this representation, the imaginary viscosity η″ (η″ = G′/ω) is plotted against the dynamic viscosity η′ (η′ = G″/ω) (The dependences of strorage (*G*′) and loss (*G*″) moduli vs. angular frequency are presented in Figure S20 in Supplementary Materials). The plot should be a perfect arc if higher order structures are absent, in which case the relaxation behavior of the melt can be described with a single relaxation time. According to Figure 9, an arc is observed for the neat PLLA and the composite PL12.

As can be seen in Figure 8 and Figure 9, the composite PL12 and neat PLLA showed similar rheological behavior. This can be attributed to a dispersive effect of PLLA-*b*-PEPA at a close to optimum [PEPA]/[pCAp] ratio; similar patterns were observed previously for HAp/PLLA [53] and CaCO_3_/PLLA [93] composites prepared with the use of oleic acid and dodecyl alcohol as compatibilizers. On the other hand, the changes of the molecular masses of PLLA during melt processing could have a bigger impact on the composite characteristics.

#### 2.2.6. Degradation of PLLA during Molding and the Role of Compatibilizer

Degradation of the polyester during melt processing greatly complicates making articles from PLLA and especially PLGA with the use of casting and 3D printing [94]; the effect of BMS on the molecular weight and dispersity of PLA during melt mixing was detected previously in a number of works [29,30,31,32,33]. Molecular weight characteristics and zero-shear viscosity values of the PLLA and composites PL07, PL08, PL11, and PL12 are given in Table 4. At higher filler content, viscosity increased (PL08 vs. PL07, PL12 vs. PL11), but more significantly, the use of 5 wt% of C1 during preparation of PL12 protected the polymer matrix from degradation.

### 2.3. Characteristics of the PCL-Based Composites

PCL is significantly inferior to PLLA and other polyesters in the strength and elastic modulus, which hampers application of PCL in bone surgery and orthopedics [95,96]. Both mechanical characteristics and bioactivity of PCL can be improved by blending with BMS [95,97]. PCL-based samples PC01–PC04 (Table 5) were prepared with 50 wt% of BMS. The temperature of molding was 90–100 °C. Among the BMS under study, pCAp is proven to be the best filler when used in PCL-based composite formulations, increasing both tensile and flexural strengths and moduli. The effect of compatibilizer C2 was also even more pronounced for PCL-based composites in comparison with PLLA: the values of σ_t_ and σ_f_ for PC04, containing 5 wt% of C2, were nearly double compared with the values of σ_t_ and σ_f_ for PCL, with a four-time increase in the moduli values. The introduction of 50 wt% BMS resulted in a marked decrease in ε values.

Thus, the addition of 50 wt% pCAp and C2 resulted in a PCL-based composite that comes close to PLLA with the values of strength and flexural moduli. There is no talk about the use of PCL/pCAp composites in cortical bone substitutes; however, a substantial improvement of strengths and moduli allows for considering pCAp as a prospective filler for the PCL matrix. Lowering of the composite elasticity seems preferred for additive manufacturing purposes, and the use of C2 compatibilizers and their analogs can substantially improve the quality of 3D-printed articles.

## 3. Materials and Methods

### 3.1. Solvents and Reagents

All solvents and reagents were supplied by Merck (Darmstadt, Germany). Toluene, diethyl ether (Et_2_O), THF, and triethylamine (NEt_3_) were refluxed with Na/benzophenone and distilled prior to use. *n*-Pentane, *n*-hexane, and *n*-heptane were refluxed for 10 h over sodium and then distilled and stored under an argon atmosphere over sodium. CH_2_Cl_2_ was refluxed over CaH_2_ and distilled. ε-Caprolactone was distilled under reduced pressure and *L*-lactide was purified with recrystallization from toluene, followed by sublimation at 0.1 Torr. 2,6-Di-*tert*-butyl-4-methylphenol (BHT, ≥99%), di-*n*-butylmagnesium (1.0 M solution in heptane), *N*,*N*-di(*n*-decyl)-*N*-methylamine, acetic acid (AcOH), ethylene glycol, PCl_3_, CaCO_3_, Na_2_CO_3_, NaHCO_3_, Na_2_HPO_4_·12H_2_O, and KH_2_PO_4_ were used as purchased.

[(BHT)Mg(μ-OBn)(THF)]_2_ (BHT-Mg) [98], 2-chloro-1,3,2-dioxaphospholane [99], and 2-*tert*-butoxy-2-oxo-1,3,2-dioxaphospholane (*t*BuOEP) [100] were synthesized as described previously (for details see Section S1 in Supplementary Materials).

For composite molding, commercial PLLA (*M*_n_ of 107 kDa and *Ð*_M_ of 2.02; supplied by FDplast, Moscow, Russia) and PCL (*M*_n_ of 88 kDa and *Ð*_M_ of 1.46; supplied by Shenzhen Bright China Industry, Shenzhen, China) were chosen as polyester components. Commercial βTCP (cat. no. 49963-10G, Sigma-Aldrich, St. Louis, MO, USA) was selected for comparison with CAp.

### 3.2. Physico-Chemical Characterization

CDCl_3_ (D, 99.8%) was supplied by Cambridge Isotope Laboratories, Inc. (Tewksbury, MA, USA). The ^1^H and ^13^C NMR spectra were recorded on a Bruker AVANCE III HD 400 spectrometer (400 MHz, Bruker Corporation, Billerica, MA, USA) at 20 °C. The chemical shifts are reported in ppm relative to the solvent residual peaks.

Scanning electron microscopy (SEM) images and energy dispersive X-ray (EDX) analysis data were obtained using a Phenom XL microscope (Thermo Fisher Scientific, Waltham, MA, USA) at an accelerating voltage of 15.0 kV.

Size exclusion chromatography (SEC) of the polymers was performed using an Agilent PL-GPC 220 chromatograph equipped with a PLgel column (Agilent Technologies, Santa Clara, CA, USA), and THF was used as the eluent (1 mL·min^−1^). The measurements were recorded with universal calibration based on a polystyrene standard at 40 °C.

X-ray diffraction (XRD) powder patterns were recorded using a MiniFLEX 600 powder diffractometer (Rigaku Corp., Tokyo, Japan) equipped with a Cu-Kα_1,2_ (λ = 1.5418 Å) X-ray tube and a 1D D/teX position-sensitive detector. Data were collected at room temperature in the 2θ range 4.5–90° with a 0.02° step size using a Bragg–Brentano setup. The cell parameters were determined with full-profile fitting, as implemented in Bruker TOPAS 5 [101].

Fourier transform infrared (FT-IR) spectra were recorded using an IFS 66 v/S spectrometer (Bruker, Billerica, MA, USA) equipped with a DLaTGS detector. Experimental parameters were the following: attenuated total reflection method, ZnSe crystal, spectral range of 600–4000 cm^−1^, resolution of 2 cm^−1^, and 15 scans.

Contact angle measurements were conducted on a LK-1 goniometer (RPC OpenScience Ltd., Krasnogorsk, Russian Federation) equipped with a Touptek-UCMOS-01300KPA digital USB camera (Hangzhou ToupTek Photonics Co., Ltd., Hangzhou, China).

Mechanical studies were carried out on a tensile machine I1140M-5-01-1 (Tochpribor-KB LLC, Ivanovo, Russian Federation). Tensile tests were conducted according to [102,103] using standard Type V dumbbell-shaped composite samples (total length of 63 mm, thickness of working part of 3.2 mm, width of 3.3 mm). The tensile rate was 1 mm·min^−1^ (PLLA-based samples) and 10 mm·min^−1^ (PCL-based samples). Bending tests were conducted according to [104] with three-point deformation of the plates (60 × 10 × 1 mm; the distance between low points of 25 mm). The flexural rate was 2 mm·min^−1^. For each composite formulation, the series of eight samples were prepared and tested. By the results of tensile tests, for each formulation, a wide part of the sample with the closest to average value of σ_t_ was sampled for thermal and rheological measurements.

Differential scanning calorimetry (DSC) experiments were performed on the TGA/DSC1 apparatus (Mettler Toledo, Columbus, OH, USA). The samples were heated from 30 °C to 200 °C (1st heating) and kept at 200 °C for 3 min to eliminate the thermal history, then cooled to 30 °C and finally heated to 200 °C again (second heating). The rate of heating and cooling was 5 K·min^−1^.

The rheological properties were investigated with a rotation stress-controlled rheometer Discovery HR-30 (TA Instruments, New Castle, DE, USA) at 175 °C using a parallel plate geometry. The plate diameter was 25 mm, while the typical gap imposed during the tests was 0.5 mm. The frequency dependences of the complex viscosity (η*) were obtained in the linear viscoelasticity region by using the variation of the angular frequency (ω) from 0.0628 to 628 rad·s^−1^. Steady shear measurements were also conducted, and the linear storage (G′) and loss (G″) moduli were measured with a strain of 1% for the frequency range of 0.001–100 Hz. The equations for calculating the rheological characteristics can be found elsewhere [105,106]; the relative error of their determination did not exceed 5%. The TRIOS 5.1.1 program package (TA Instruments, New Castle, DE, USA) was used for the treatment and presentation of the results.

The degree of crystallinity *χ*_c_ for PLLA-based composites was calculated from the difference between Δ*H*_m_ and Δ*H*_c_ values for the sample and Δ*H*_m_ of 100% crystalline PLLA (Equation (2)),

(2) $$\chi_{C}=\frac{\Delta H_{m}-\Delta H_{c}}{93}\times 100\%,$$

where 93 is Δ*H*_m_ of 100% crystalline PLLA (J·g^−1^) [32].

### 3.3. Synthesis of Copolymers

#### 3.3.1. BnO(^t^BuOEP)_23_-b-(L-LA)_142_ (Copolymer C1)

*^t^*BuOEP (0.85 g, 4.7 mmol) and CH_2_Cl_2_ (3.0 mL) were placed under an argon atmosphere into a flame-dried flask. A solution of [(BHT)Mg(μ-OBn)(THF)]_2_ (0.1 g, 0.24 mmol) in CH_2_Cl_2_ (1.0 mL) was added. After 2 h of stirring at 20 °C, a solution of *L*-lactide (2.73 g, 19 mmol) in CH_2_Cl_2_ (4.5 mL) was added, and the mixture was stirred within 12 h. AcOH (14 mg, 0.5 mmol) was added. The polymer was precipitated using a 10-fold volume excess of dry Et_2_O, and dissolution in CH_2_Cl_2_ and precipitation was repeated three times. The yield was 2.46 g (67%).

^1^H NMR (400 MHz, CDCl_3_, 20 °C): δ 7.37 (m, 5H, aromatic ring); 5.16 (q, ^3^*J* = 7.1 Hz, 286H, C*H*CH_3_); 5.04 (d, 2H, PhC*H*_2_); 4.18 (m, 92H, POC*H*_2_); 1.57 (d, ^3^*J* = 7.1 Hz, 844H, CHC*H*_3_); 1.50 (s, 224H). ^31^P{^1^H} NMR (162 MHz, CDCl_3_, 20 °C): δ −5.48 (1P); −5.65 (23P).

Comparison of the integral intensities of the signals of BnO end-groups and comonomer fragments in the ^1^H NMR spectrum (Figure S2 in Supplementary Materials), and the signals of the phosphorus atoms in main-chain and end fragments in the ^31^P NMR spectrum (Figure S3 in Supplementary Materials), allowed us to determine the composition of C1 as BnO(*^t^*BuOEP)_23_-*b*-(*L*-LA)_142_.

#### 3.3.2. BnO(εCL)_119_-*b*-(*^t^*BuOEP)_24_ (Copolymer C2)

ε-Caprolactone (1.08 g, 9.5 mmol) and CH_2_Cl_2_ (4.0 mL) were placed under an argon atmosphere into a flame-dried flask. A solution of [(BHT)Mg(μ-OBn)(THF)]_2_ (50 mg, 0.12 mmol) in CH_2_Cl_2_ (0.5 mL) was added. After 90 min of stirring at 20 °C, *^t^*BuOEP (0.43 g, 2.4 mmol) was added, and the mixture was stirred within 12 h. AcOH (7.0 mg, 0.25 mmol) was added. The polymer was precipitated using a 10-fold volume excess of dry Et_2_O, and dissolution in CH_2_Cl_2_ and precipitation was repeated three times. The yield was 1.15 g (74%).

^1^H NMR (400 MHz, CDCl_3_, 20 °C): δ 7.32 (m, 5H, aromatic ring); 5.08 (s, 2H, PhC*H*_2_); 4.16 (m, 96H, POC*H*_2_); 4.03 (t, ^3^*J* = 6.8 Hz, 238H); 2.28 (t, ^3^*J* = 7.4 Hz, 236H); 1.62 (m, 476H); 1.48 (s, 214H, *^t^Bu*); 1.35 (m, 240H). ^31^P{^1^H} NMR (162 MHz, CDCl_3_, 20 *°*C): δ −5.42 (1P); −5.69 (24P).

The composition of C2 was determined as BnO(εCL)_119_-*b*-(*^t^*BuOEP)_24_ with the analysis of ^1^H and ^31^P NMR spectra (Figures S4 and S5 in Supplementary Materials).

### 3.4. Synthesis of CAp

#### 3.4.1. Synthesis of hCAp

CaCO_3_ (100.1 g, 1 mol) and Na_2_[EDTA]·2H_2_O (372 g, 1 mol) were dissolved in H_2_O (1 L). Then, Na_2_HPO_4_·12H_2_O (215 g, 0.6 mol) and Na_2_CO_3_ (106 g, 1 mol) were added. After 1 h at 100 °C, the mixture was filtered to a 1.8 L autoclave placed in a heating bath. After the addition of water (total volume, 1.6 L), the autoclave was heated at 150 °C within 5 h. After cooling to 20 °C, the precipitate was separated with decantation, washed with H_2_O (2 × 300 mL), ^i^PrOH (2 × 200 mL), and pentane (2 × 100 mL), and dried in vacuo. The yield was 16.74 g (18.6%).

#### 3.4.2. Synthesis of pCAp

CaCO_3_ (100.1 g, 1 mol) and Na_2_[EDTA]·2H_2_O (372 g, 1 mol) were dissolved in H_2_O (1 L). The solution was filtered to a 1.8 L autoclave and pre-loaded with NaHCO_3_ (84.06 g, 1 mol) and KH_2_PO_4_ (81.65 g, 0.6 mol). After the addition of water (total volume, 1.6 L), the autoclave was heated at 140 °C within 24 h. After cooling to 20 °C, the precipitate was separated with decantation, washed with H_2_O (2 × 300 mL), ^i^PrOH (2 × 200 mL), and pentane (2 × 100 mL), and dried in vacuo. The yield was 18.70 g (19.6%).

### 3.5. Preparation of the Composites and Composite Samples

#### 3.5.1. PLLA-Based Composites

PLLA (7 g) was dissolved in THF (50 mL). BMS (calculated amount) was added. After 1 h of stirring at 20 °C, the mixture was poured into a PTFE cuvette and dried at 100 °C within 12 h. In the presence of C1, the stirring of the reaction components (BMS and C1) was conducted in a closed glass vial at 90 °C within 1 h. Cooled suspension was added to the solution of PLLA in THF (20 mL). After 1 h of stirring at 20 °C, the mixture was poured into the PTFE cuvette and dried at 100 °C within 12 h.

#### 3.5.2. PCL-Based Composites

PCL (7 g) was dissolved in THF (50 mL). BMS (calculated amount) was added. After 1 h of stirring at 20 °C, the mixture was poured into a PTFE cuvette and dried at 50 °C within 2 days. In the presence of C2, the stirring of the reaction components (BMS and C2) was conducted in a closed glass vial at 90 °C within 1 h. Cooled suspension was added to the solution of PCL in THF (20 mL). After 1 h of stirring at 20 °C, the mixture was poured into the PTFE cuvette and dried at 50 °C within 2 days.

#### 3.5.3. Molding of the Composite Samples

Melt homogenization of the composites was carried out using a Polydrive mixer HAAKE R600 (Thermo Electron Scientific, Madison, WI, USA). Before mixing, the composite films were shredded manually and dried at 50 °C and 0.01 Torr within 12 h. Molding of the samples was made on HAAKE MINIJet Pro equipment (Thermo Fisher Scientific, Waltham, MA, USA) with the pressure of 600 Bar. For PLLA-based composites, the temperatures of molding were 180–200 °C (cylinder) and 65 °C (mold), for PCL-based composites, 70–90 °C (cylinder) and 30 °C (mold). A Mould Tensile Bar ASTM 0638-V injection mold was used for preparation of the samples for a tensile test (Figure S13 in Supplementary Materials), and a Mould HAAKE MiniJet 60 × 10 × 1 injection mold was used for casting of the samples for a bending test.

### 3.6. Statistical Analysis

The results of the mechanical tests are presented as the mean ± standard deviation (SD) and median with the interquartile range. The data were analyzed with SigmaStat 3.5 software (Systat Software Inc., San Jose, CA, USA).

## 4. Conclusions

In the present work, we assumed that the properties of polyester/BMS composites can be substantially improved when using polymeric compatibilizers comprising long polyester and a phosphorus-containing polyacid block. Having chosen PLLA and PCL as polyester components, we prepared PLLA/BMS composites with 10–60 wt% loading of the inorganic filler and PCL-based composites with 50 wt% of BMS. Micro-sized commercial βTCP and synthesized carbonated apatites hCAp and pCAp were used as BMS. The synthesis of hCAp and pCAp was based on our previous study [47] but was substantially optimized to achieve reproducibility at a tens of grams level. Reactive copolymers BnO(*^t^*BuOEP)_23_-*b*-(*L*-LA)_142_ (C1) and BnO(εCL)_119_-*b*-(*^t^*BuOEP)_24_ (C2) were synthesized and used as compatibilizer precursors.

At the first stage of the composite preparation, all components were dispersed in THF media, and additional heating at 90 °C resulted in formation of polyester-*b*-PEPA compatibilizers. Tensile and bending tests of the molded PLLA-based samples showed the better mechanical characteristics for pCAp-based formulations, viz. a lower decrease in σ_t_ and σ_f_ values, as well as a higher increase in *E*_t_ and *E*_f_ values. An additional improvement of the pCAp-based composite properties was achieved with the addition of 5 wt% of C1. The best set of mechanical characteristics was demonstrated by the sample PL12, prepared with the use of C1: even with the 25 wt% filler content, the material exceeded neat PLLA in strength and moduli values. The positive impact of the use of C2 on the mechanical characteristics of the PCL/pCAp composite was even more pronounced; the composite with 50 wt% of the filler had nearly doubled strength values relative to σ_t_ and σ_f_ of PCL, with an almost four-time increase in moduli.

The effect of C1 and C2 on composite properties was attributed to the formation of polyester-*b*-PEPA copolymers followed by chemical bonding between the PEPA block and pCAp surface. This bonding was confirmed with FT-IR studies of the mineral residues after dissolution and elimination of the PLLA matrix from the samples PL09 and PL13, prepared without and with the addition of C1; in the latter case, the presence of PLLA, chemically bonded with the pCAp surface, was confirmed by the presence of characteristic signals at ~1800 (>C=O) and 1220 (C–O) cm^−1^. SEM data for PL13 showed that during composite molding, PEPA-*b*-PLLA served as an efficient dispersant for pCAp splices, whose structure remained unchanged during preparation of PL09.

The use of C1 provided a more pronounced nucleation effect of the filler during PLLA crystallization (SEC data). Additional experiments on isothermal crystallization at 110 °C showed the immediate onset of crystallization only for samples PL12 and PL13 (25 and 50 wt% of the filler) prepared with the addition of C1. Rheological studies at 175 °C showed a significant reduction in viscosity in the case of pCAp-containing composites that can be attributed to a decrease in the molecular weight of PLLA components. The decline in MW can be caused by the presence of BMS and their impact on thermal degradation of PLLA, and the presence of a compatibilizer could prevent or weaken the effect of the filler. We observed a similar weakening in the example of PL12 with 25 wt% content of pCAp; during melt processing, *M*_w_ of PLLA in this composite was 12% lower relative to *M*_w_ of neat PLLA, whereas in the absence of the compatibilizer, the loss of MW was 35%.

PLLA-based composite samples, obtained during this study, do not reach the mechanical characteristics of the cortical bone [10,11] but outperform the characteristics of trabescular bone [107]. However, the effect of BMS with appropriate morphology, especially in combination with PEPA-containing compatibilizers, shifts the properties of the composite to higher values of strengths and moduli. The further development of polyester/BMS composites can be based on the replacement of PLLA or PCL, used in this work, by PLGA or alternative biodegradable polyesters with improved basic mechanical characteristics [35]. Combination of selected polyesters, perfectly shaped plate-like CAp, and PEPA-containing compatibilizers with a proven BM affinity [70,71,72,73,74] and osteoinductivity [76,77], as well as dispersive and protective properties, demonstrated in the present work, would be able to provide the further development of BMS-based composites and articles therefrom, prepared with hot melt processing.

## Figures and Tables

**Table 1:** Crystal cell parameters and chemical composition of hCAp and pCAp.

CAp Type	Crystal Cell Parameters, XRD		CSR, nm ^1^	wt% CO_3_^2−^		C/P Ratio ^3^	Na/Ca Ratio ^3^
	*a*, Å	*c*, Å		FT-IR Data	TGA Data ^2^		
hCAp	9.412	6.944	>1000	12.1	9.6	0.42	0.18
pCAp	9.430	6.915	>1000	4.9	4.0	0.19	0.08

^1^ Coherent scattering region. ^2^ Mass loss in the temperature range 400–1000 °C. ^3^ EDX data.

**Table 2:** Composition and mechanical characteristics of PLLA/BMS samples with average squared deviations.

Comp. Sample	Addition of C1, 5 wt%	BMS	BMS wt%	Tensile Test			Flexural Test	
				ε, %	σ_t_, MPa	*E*_t_, GPa	σ_f_, MPa	*E*_f_, GPa
PLLA	−	no filler	0	2.46 ± 0.33	53.2 ± 1.7	2.18 ± 0.15	99.7 ± 5.6	3.31 ± 0.32
PL01	−	βTCP	10	3.37 ± 0.24	47.0 ± 0.4	2.26 ± 0.11	92.3 ± 1.9	3.70 ± 0.13
PL02	−	βTCP	25	2.60 ± 0.16	46.1 ± 1.1	2.45 ± 0.12	91.0 ± 1.9	4.47 ± 0.09
PL03	−	βTCP	50	1.69 ± 0.05	43.6 ± 0.9	2.81 ± 0.15	80.9 ± 1.2	5.86 ± 0.15
PL04	−	hCAp	10	3.44 ± 0.21	41.8 ± 1.1	2.33 ± 0.18	93.3 ± 2.1	3.55 ± 0.12
PL05	−	hCAp	25	3.10 ± 0.81	36.0 ± 1.2	2.57 ± 0.26	87.9 ± 1.8	4.49 ± 0.15
PL06	−	hCAp	50	1.33 ± 0.12	33.5 ± 1.0	2.86 ± 0.20	76.3 ± 0.8	5.76 ± 0.11
PL07	−	pCAp	10	2.54 ± 0.19	47.8 ± 1.6	2.40 ± 0.09	94.5 ± 1.8	4.11 ± 0.09
PL08	−	pCAp	25	1.64 ± 0.16	49.1 ± 2.0	2.78 ± 0.17	88.8 ± 4.1	5.49 ± 0.06
PL09	−	pCAp	50	1.27 ± 0.06	47.3 ± 0.8	3.29 ± 0.18	79.3 ± 5.2	9.03 ± 0.38
PL10	−	pCAp	60	1.08 ± 0.07	42.2 ± 3.6	3.42 ± 0.18	64.1 ± 13.1 ^1^	10.12 ± 0.85
PL11	+	pCAp	10	3.52 ± 0.91	52.6 ± 3.4	2.53 ± 0.27	92.6 ± 3.6	7.70 ± 0.52
PL12	+	pCAp	25	2.54 ± 0.14	62.2 ± 1.4	2.69 ± 0.11	93.8 ± 3.1	8.09 ± 0.33
PL13	+	pCAp	50	1.54 ± 0.09	52.9 ± 1.3	3.08 ± 0.06	82.6 ± 4.4	9.51 ± 0.74
PL14	+	pCAp	60	1.34 ± 0.02	51.4 ± 1.6	3.35 ± 0.18	75.4 ± 3.7	11.22 ± 0.62

^1^ Fragile material, low reproducibility of the measurements.

**Table 3:** Thermal properties of PLLA-based composites ^1^.

Comp. Sample	1st Heating							Cooling after 1st Heating	
	*T*_g_, °C	Δ*C*_p_^2^, J·g^−1^·K^−1^	*T*_c_, °C	Δ*H*_c_^2^, J·g^−1^	*T*_m_, °C	Δ*H*_m_^2^, J·g^−1^	χ_c_^3^, %	*T*_g_, °C	ΔCp ^2^, J·g^−1^·K^−1^
PLLA	62.2	0.55	118.4	23.5	154.3	25.0	1.6	55.5	0.51
PL02	58.6	0.52	108.7	24.6	153.0	27.6	3.2	55.1	0.55
PL05	55.8	0.48	103.5	23.7	151.3	24.9	1.1	55.4	0.49
PL07	56.4	0.35	109.2	27.7	155.3	30.9	3.6	54.5	0.47
PL08	56.9	0.48	107.2	23.5	154.7	31.0	8.0	54.8	0.50
PL09	58.6	0.50	108.1	23.6	153.1	25.6	4.3	53.7	0.49
PL11	52.9	0.41	102.4	24.3	154.8	30.9	7.1	54.8	0.53
PL12	56.6	0.33	107.9	21.8	155.0	29.2	7.9	54.3	0.59
PL13	57.3	0.24	108.5	24.9	154.9	29.7	5.1	54.6	0.42

^1^ Heating/cooling rate, 5 K·min^−1^. ^2^ The values of Δ*C*_p_, Δ*H*_c_, and Δ*H*_m_ are normalized, taking into account wt% of PLLA in composite. ^3^ Degree of crystallinity.

**Table 4:** Molecular weight characteristics and zero-shear viscosity of PLLA and PLLA/pCAp composites.

Composite	*M*_w_	*M*_n_	*Ð*_M_	*η*_0_, Pa·s
PLLA	173.62	90.54	1.92	1694
PL07	111.32	45.69	1.97	124
PL08	113.42	58.75	1.93	561
PL11	121.23	61.68	2.44	212
PL12	153.38	78.10	1.96	1304

**Table 5:** Composition and mechanical characteristics of PCL/BMS samples with average squared deviations.

Comp. Sample	Addition of C1, 5 wt%	BMS	BMS wt%	Tensile Test			Flexural Test	
				ε, %	σ_t_, MPa	*E*_t_, GPa	σ_f_, MPa	*E*_f_, GPa
PCL	−	no filler	0	>100 ^1^	15.0 ± 0.8	0.46 ± 0.03	23.7 ± 2.0	0.66 ± 0.06
PC01	−	βTCP	50	16.8 ± 2.8	18.4 ± 1.0	1.53 ± 0.04	26.4 ± 1.2	1.15 ± 0.09
PC02	−	hCAp	50	17.6 ± 2.6	15.2 ± 1.4	1.22 ± 0.05	24.4 ± 1.4	1.25 ± 0.03
PC03	−	pCAp	50	6.2 ± 0.8	16.5 ± 0.4	1.48 ± 0.06	37.2 ± 1.0	2.55 ± 0.04
PC04	+	pCAp	50	8.1 ± 0.8	28.3 ± 0.6	1.85 ± 0.15	41.4 ± 1.7	2.67 ± 0.21

^1^ 100% elongation is a limit for the equipment used in this work (see Section 3.2).

## Data Availability

The data presented in this study are available on request from the corresponding author.

## Supplementary Materials

The following supporting information can be downloaded at: https://www.mdpi.com/article/10.3390/ijms241311175/s1. References [108,109,110,111,112] are cited in the Supplementary Materials.
