# Supplementary material for: Dispersant and Protective Roles of Amphiphilic Poly(ethylene phosphate) Block Copolymers in Polyester/Bone Mineral Composites
Source: Int J Mol Sci. 2023 Jul 6;24(13):11175. doi: 10.3390/ijms241311175 (PMC10342518; doi:10.3390/ijms241311175)
Supplement: Supplementary file 1 [file ijms-24-11175-s001.zip › ijms-2481242-supplementary.pdf]

Dispersant and Protective Roles of Amphiphilic Poly(ethylene phosphate) Block Copolymers in Polyester/Bone Mineral Composites

Ilya Nifant'ev [1,2,3,*], Alexander Tavtorkin [1], Pavel Komarov [1], Egor Kretov[1,3], Sofia Korchagina[1], Maria Chinova [1], Dmitry Gavrilov [1,2], Pavel Ivchenko [1,2]

[1] A.V. Topchiev Institute of Petrochemical Synthesis RAS, 29 Leninsky Pr., 119991 Moscow, Russia; tavtorkin@yandex.ru (A.T.); komarrikov@yandex.ru (P.K.); egor.kretov.02@mail.ru (E.K.); korchagina@ips.ac.ru (S.K.); chinova@yandex.ru (M.C.); gavrosdm@gmail.com (D.G.); phpasha1@yandex.ru (P.I.)

[2] Chemistry Department, M.V. Lomonosov Moscow State University, 1–3 Leninskie Gory, 119991 Moscow, Russia

[3] Faculty of Chemistry, National Research University Higher School of Economics, Myasnitskaya St. 20, 101100 Moscow, Russia

* Correspondence: inif@org.chem.msu.ru

Supplementary Information

S1. Copolymers 2

S2. Characteristics of BMS 5

S3. Preparation and testing of the composites 11

S1. Copolymers

S1.1. Synthesis and purification of ^{t}BuOEP

The synthesis of ^{t}BuOEP was conducted as described previously [1,2]. Since controlled preparation of block copolymers requires high purity of the cyclic monomers, the method of purification of ^{t}BuOEP was modified: after separation of *m*-chlorobenzoic acid and elimination of the solvent under reduced pressure, *N*,*N*-di(*n*-decyl)-*N*-methylamine (5 wt%) was added to the residue, and ^{t}BuOEP was distilled with external heating by heat gun (150 °C) at 0.03 Torr. The yields were 45–55%, depending on the reagent loading. ^{1}H NMR spectrum of ^{t}BuOEP (Figure S1) confirms sufficient purity of ^{t}BuOEP.

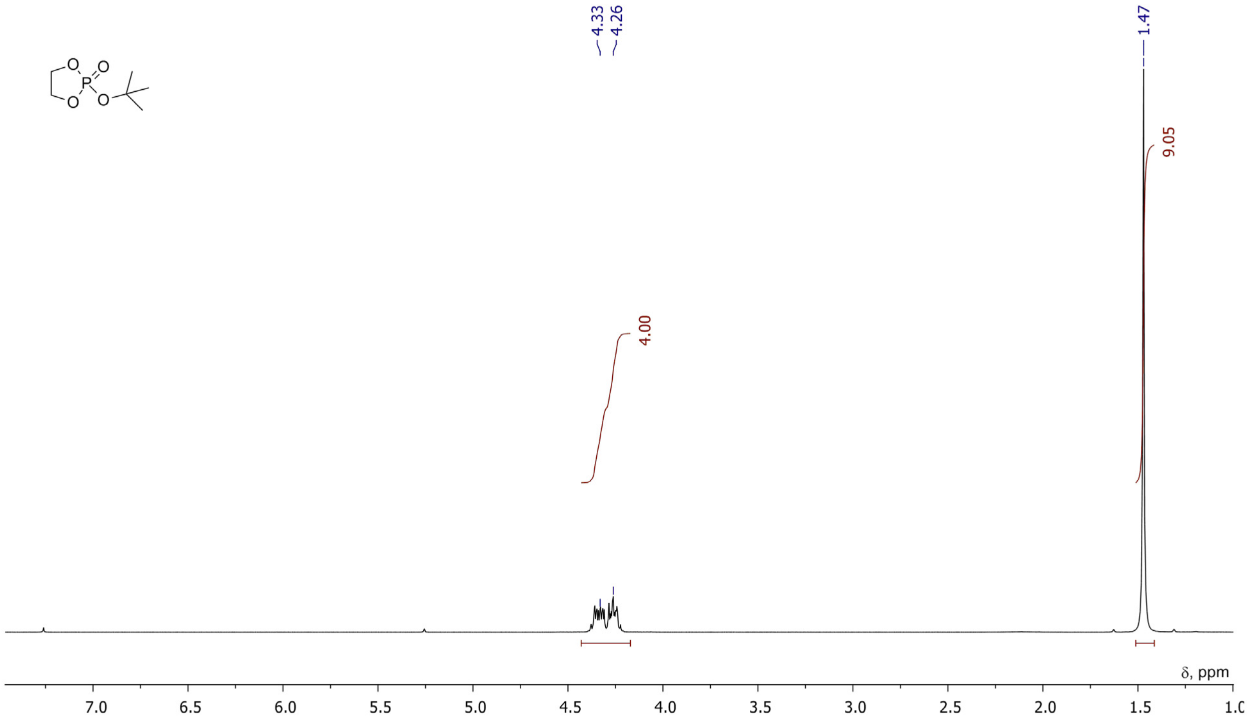

Figure S1. ^{1}H NMR spectrum ($CDCl_3$, 20 °C, 400 MHz) of ^{t}BuOEP.

Other comonomers, *L*-lactide and ε-caprolactone, also required additional purification (see Section 2.1).

S1.2. NMR spectra of copolymer C1

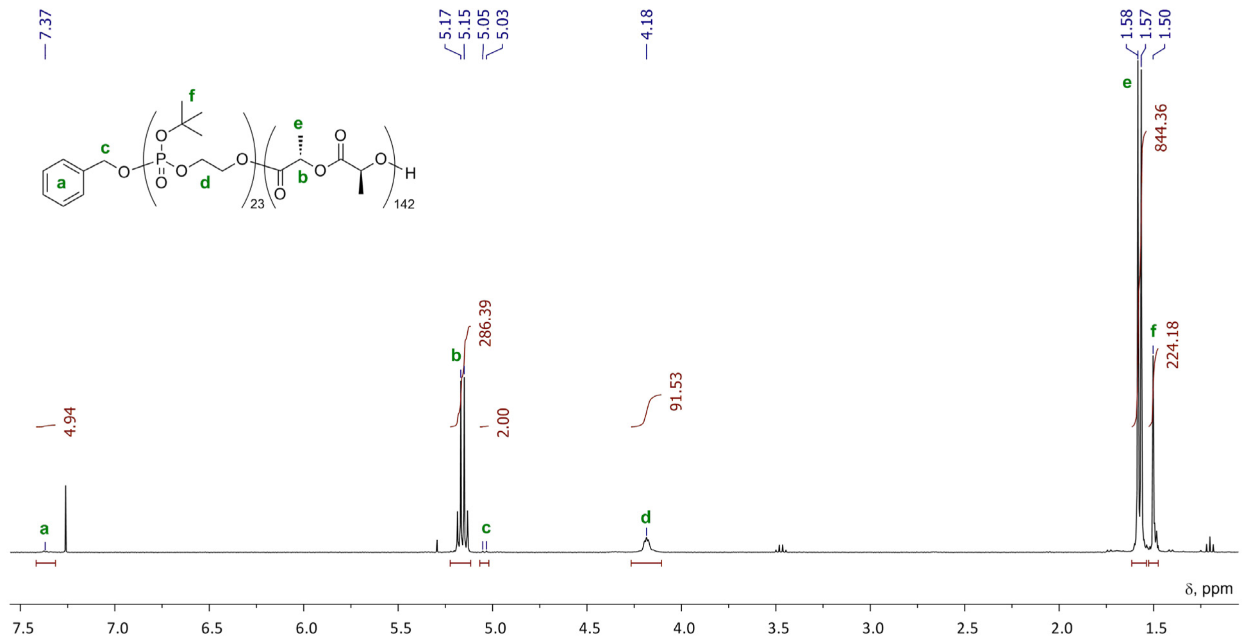

Figure S2. ^{1}H NMR spectrum ($CDCl_3$, 20 °C, 400 MHz) of C1.

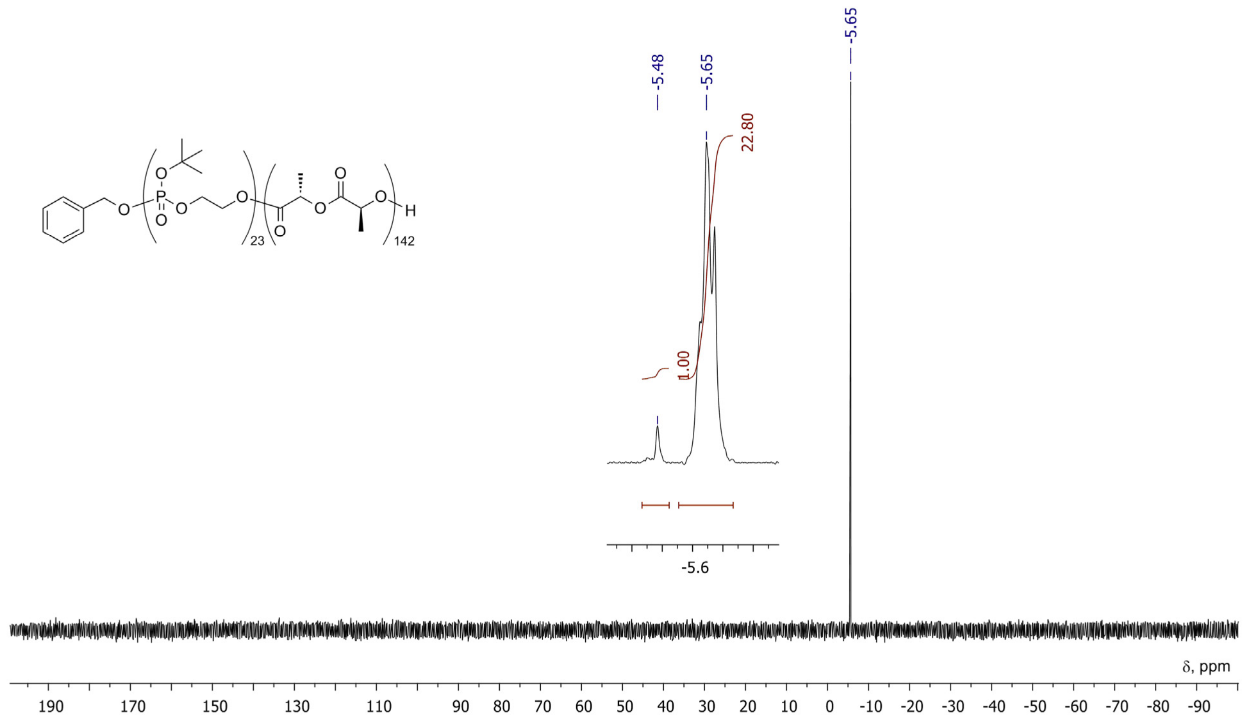

Figure S3. ^{31}P NMR spectrum ($CDCl_3$, 20 °C, 162 MHz) of C1.

S1.3. NMR spectra of copolymer C2

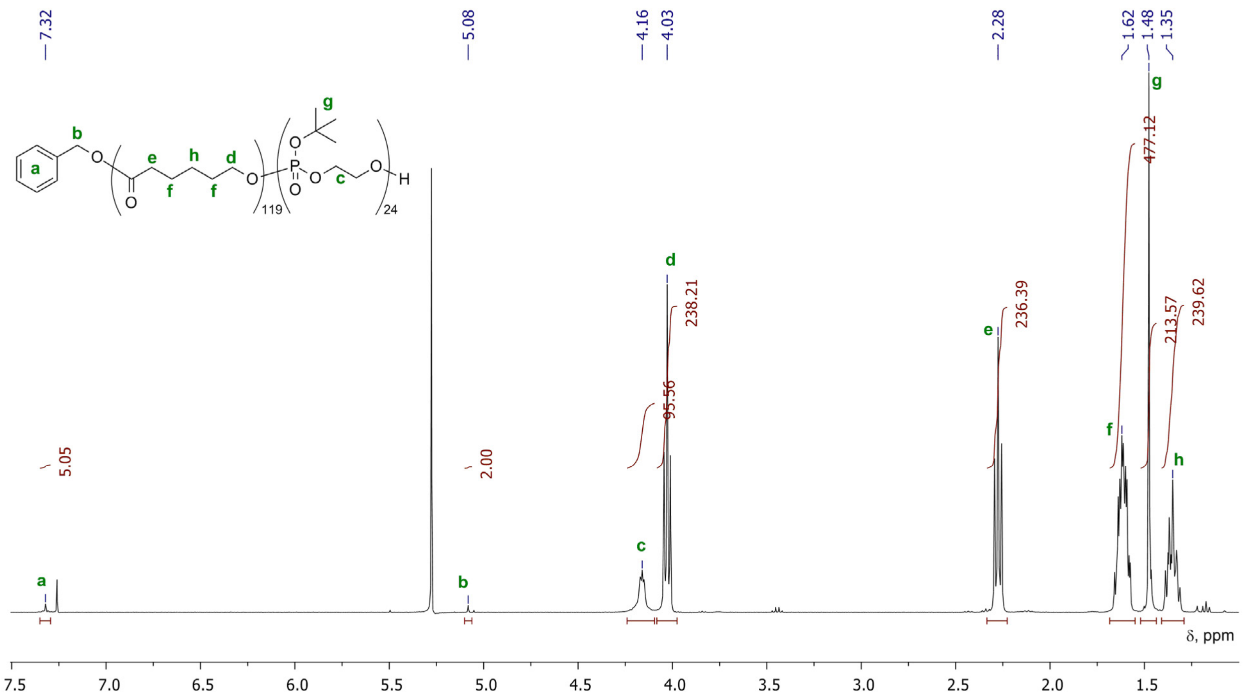

Figure S4. ^{1}H NMR spectrum ($CDCl_3$, 20 °C, 400 MHz) of C2.

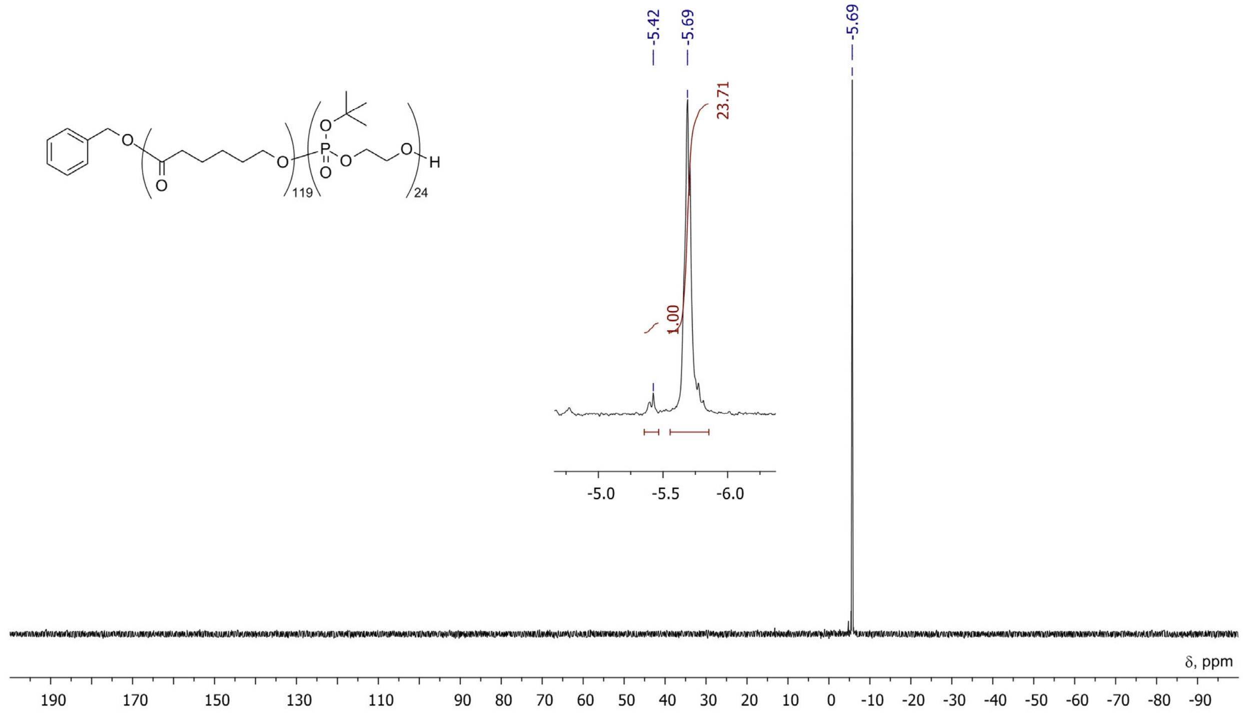

Figure S5. ^{31}P NMR spectrum ($CDCl_3$, 20 °C, 162 MHz) of C2.

S2. Characteristics of BMS

S2.2. SEM images of BMS

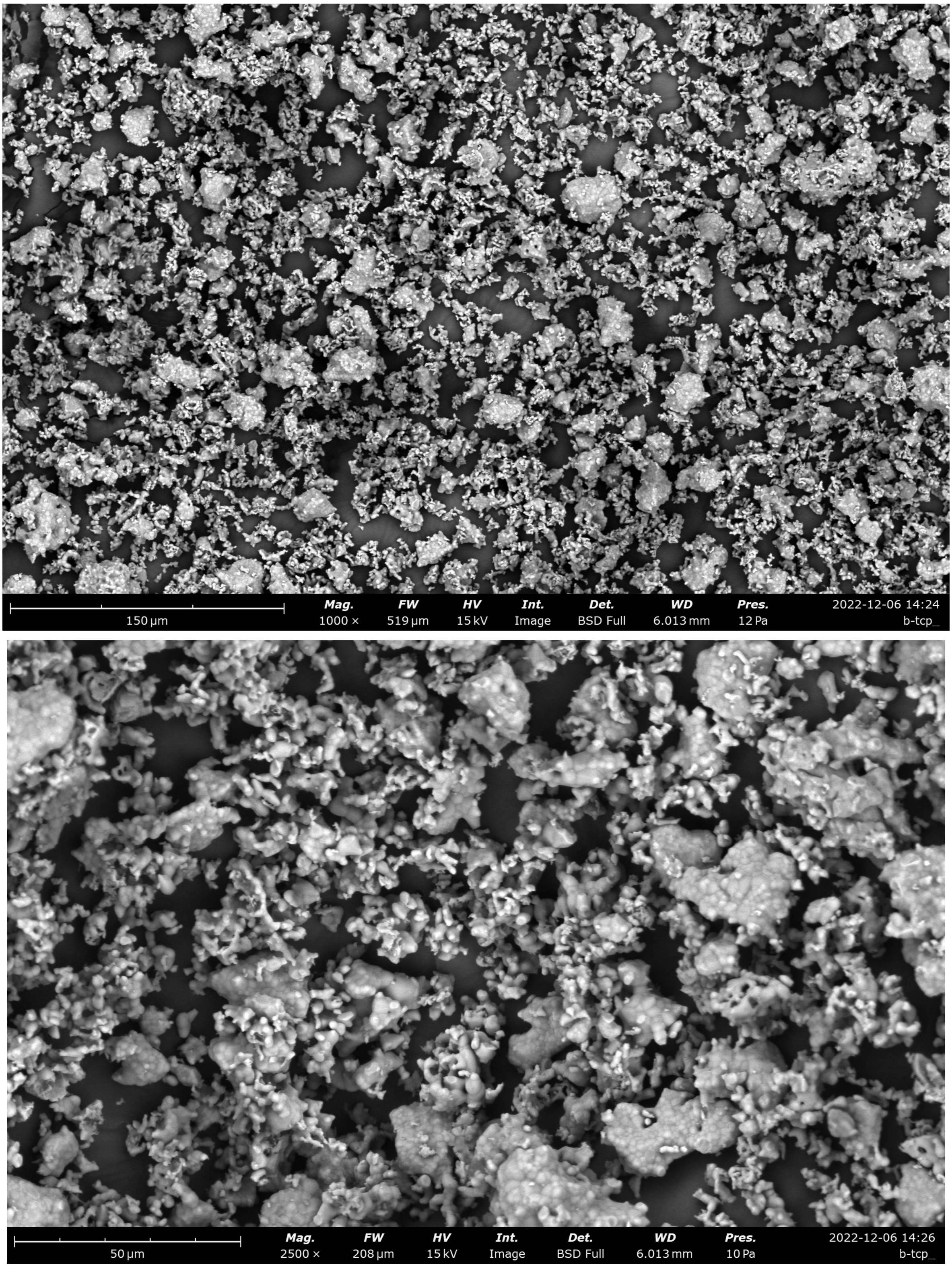

Figure S6. SEM images of βTCP.

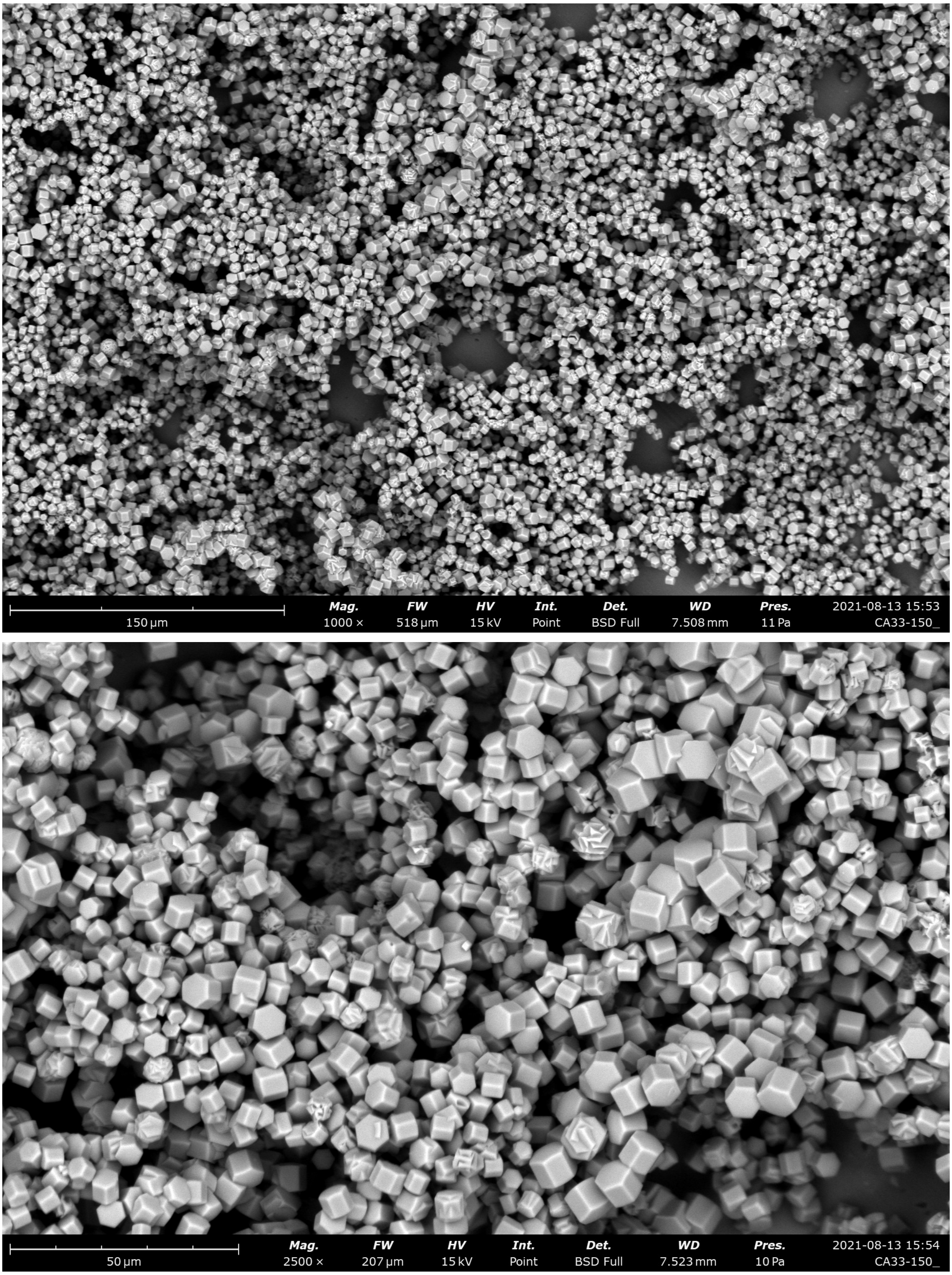

Figure S7. SEM images of hCAp.

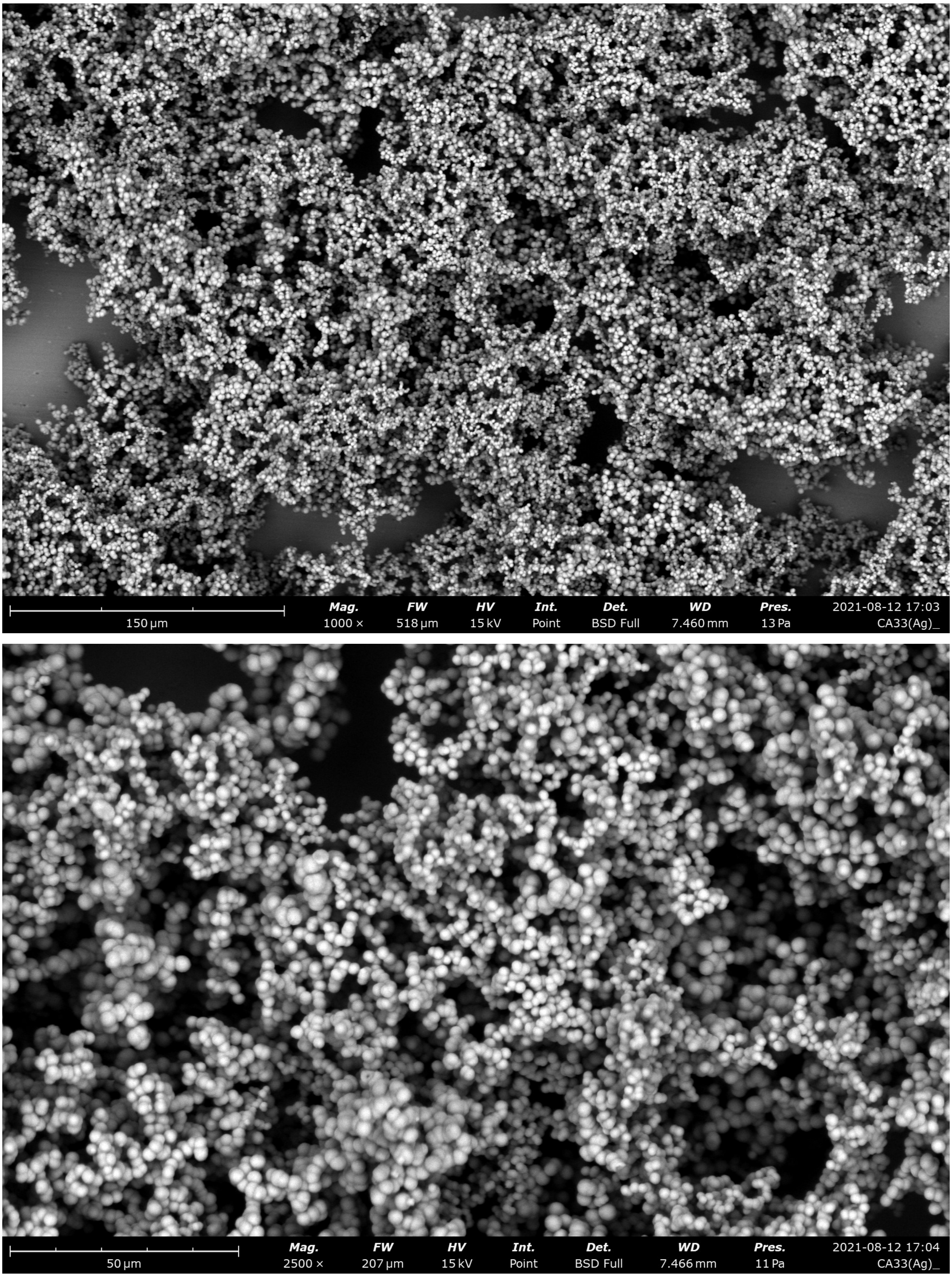

Figure S8. SEM images of intermediate phase formed during the synthesis of hCAp.

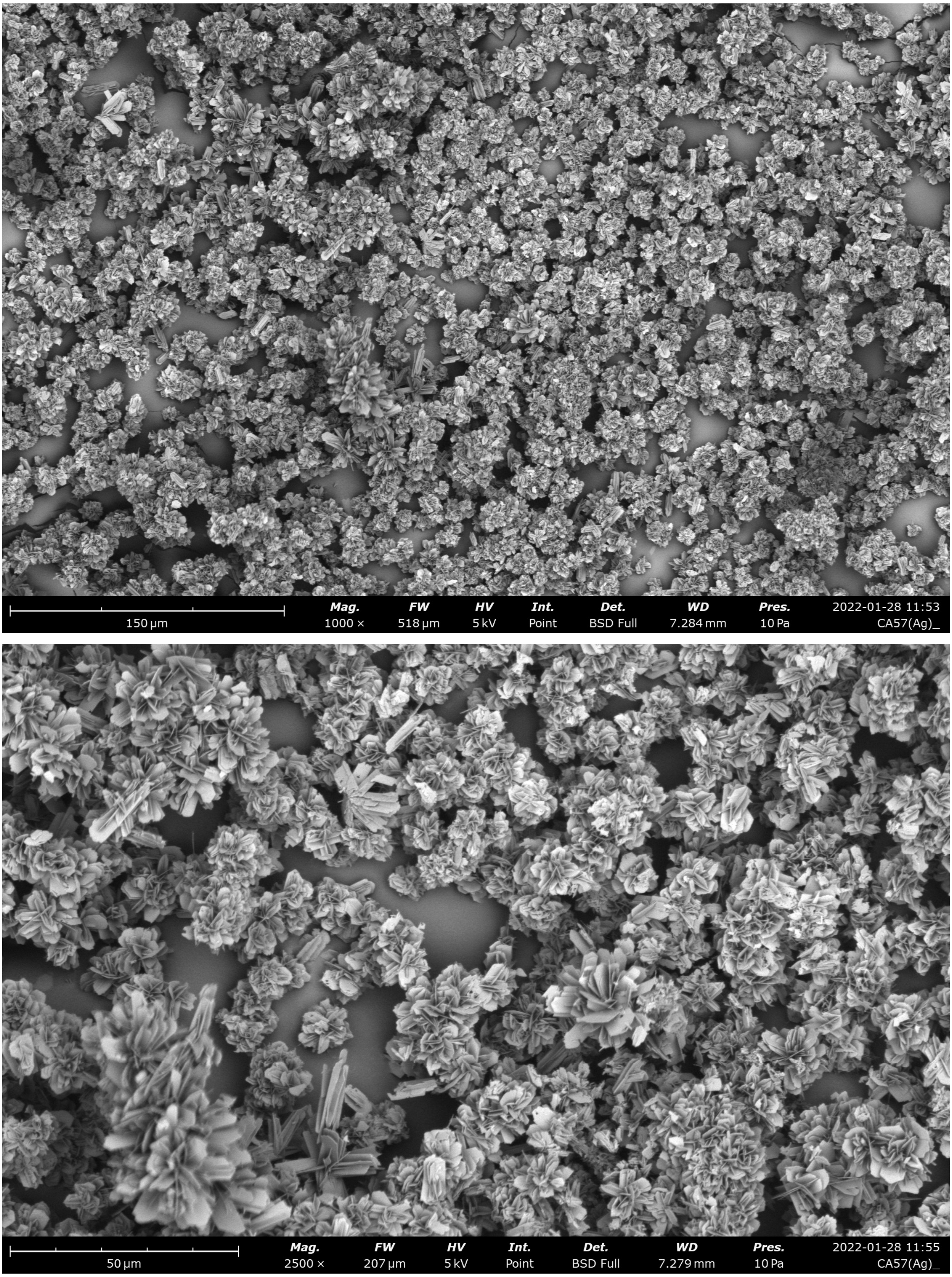

Figure S9. SEM images of pCAp.

S2.2. XRD analysis of BMS

During the study of CAp samples, cell parameters were determined by Pawley refinement. Experimental (blue) and calculated (red) diffractograms, difference curves (gray) and impurity peaks (vertical lines) are presented in figures below. Both samples belong to hydroxyapatite family, however, crystal cell parameters *a* and *c* differ substantially.

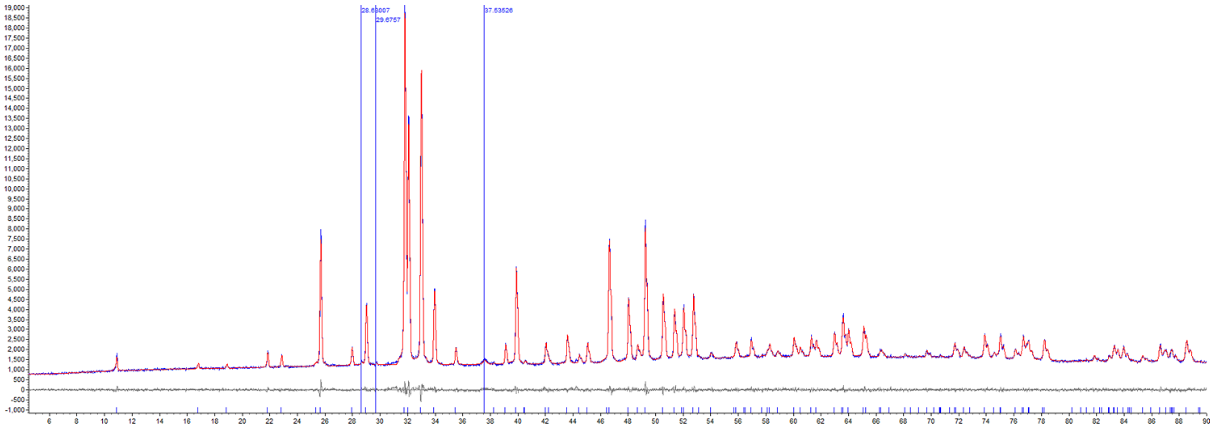

Figure S10. XRD patterns of hCAp.

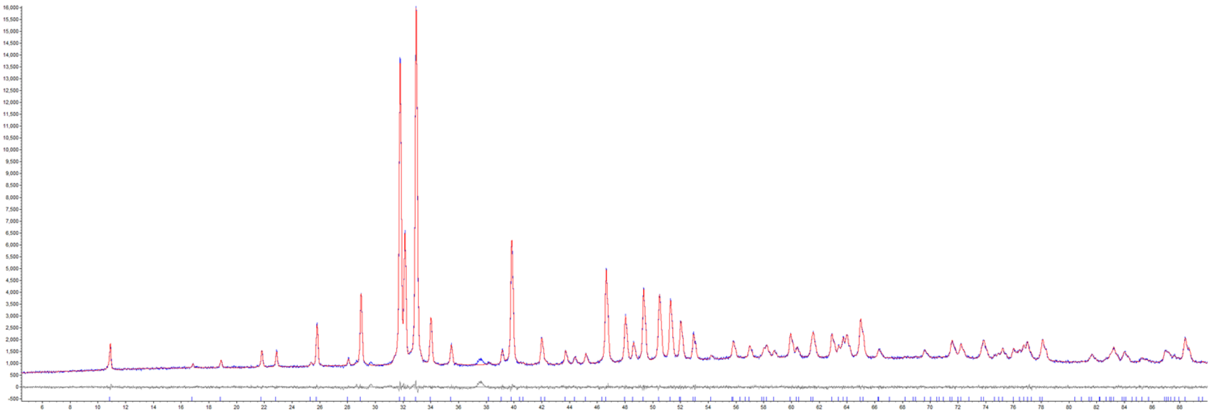

Figure S11. XRD patterns of pCAp.

S2.3. FT-IR spectra of BMS

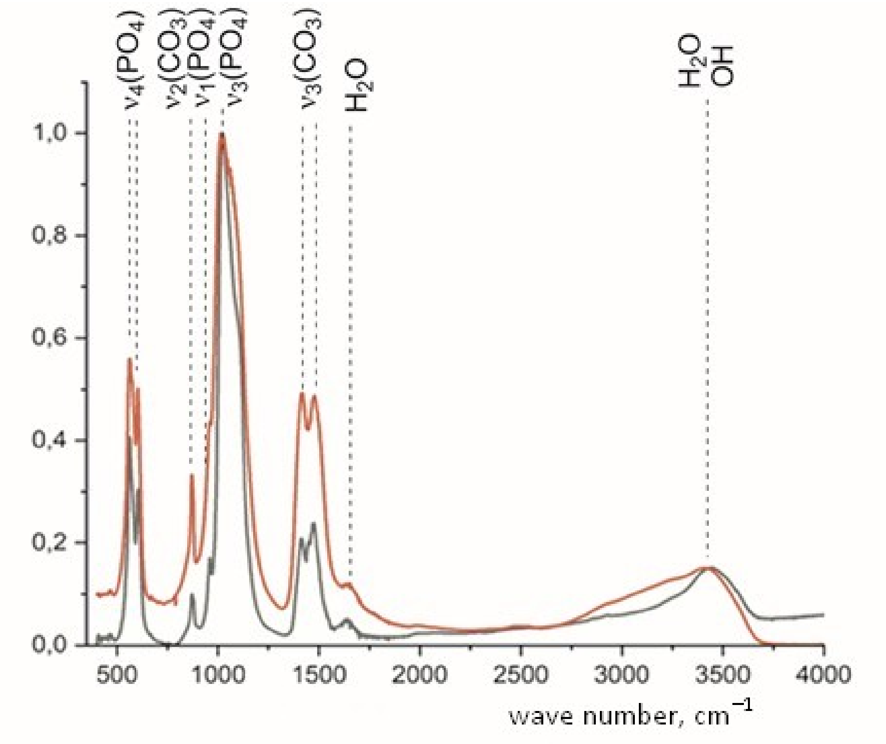

Figure S12. FT-IR spectra of hCAp (red) and pCAp (black).

The substitution of PO_4^{3-} and OH^- by CO_3^{2-} in HAp with and without Na^+ incorporation was detected by FT-IR spectroscopy. As reported previously [3–6], the characteristic bands for carbonate occur in the spectral regions 1400–1600 cm^{-1} (ν_3; asymmetric stretching vibration) and 873–880 cm^{-1} (ν_2; out-of-plane bending vibration). In addition, a weak band for the stretching vibration of structurally bound OH^- may be present in CAp samples near 3572 cm^{-1}. A combined experimental and computational study revealed that the carbonate ν_3 bands at 1546 and 1465 cm^{-1} are IR signatures for A-type and B-type CAp, respectively [7].

S3. Preparation and testing of the composites

S3.1. Preparation and mechanical testing of the samples

During tensile tests, composite-molded samples have demonstrated low elongation at break (**ε**) values (e. g. Figure S13).

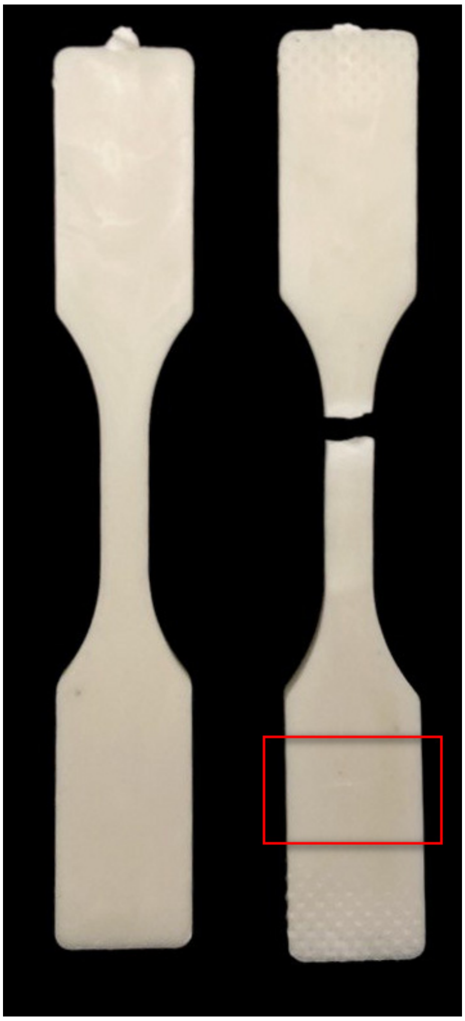

Figure S13. Samples of PL12 before (left) and after (right) tensile test. The sample fragment, used for thermal and mechanical tests, is marked in red.

S3.2. SEM imaging of the samples

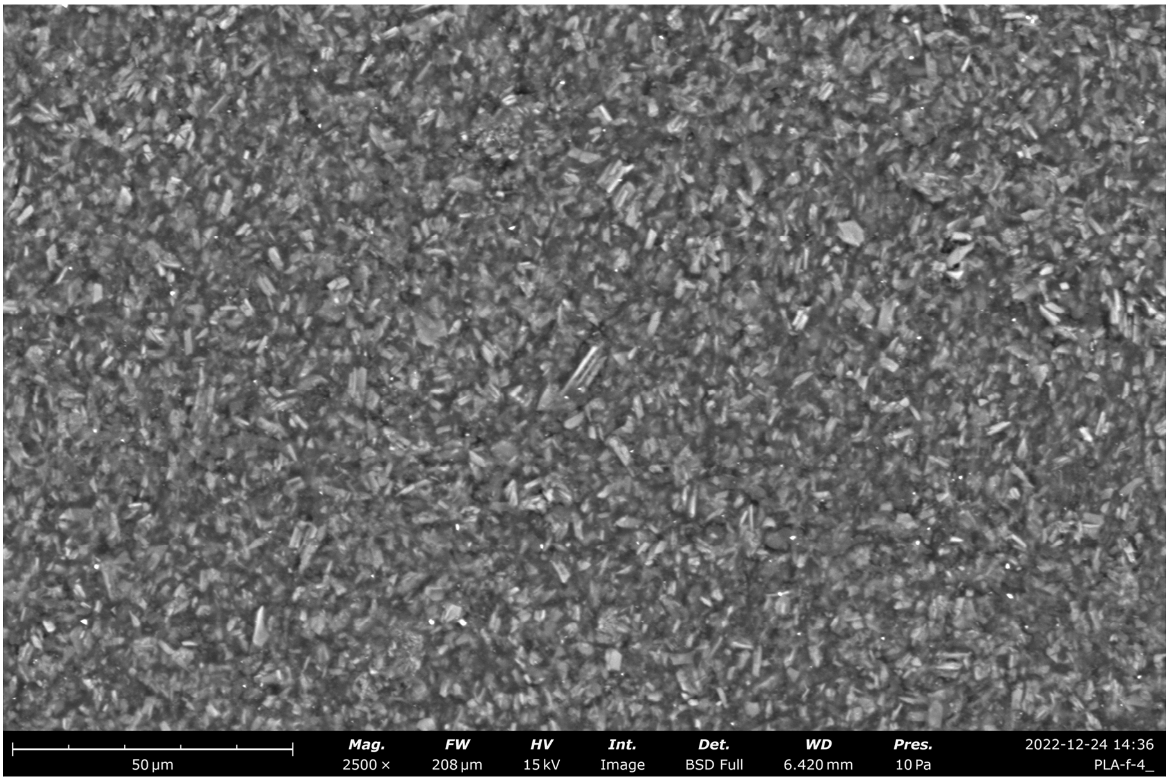

Figure S14. SEM image of the surface of PL09.

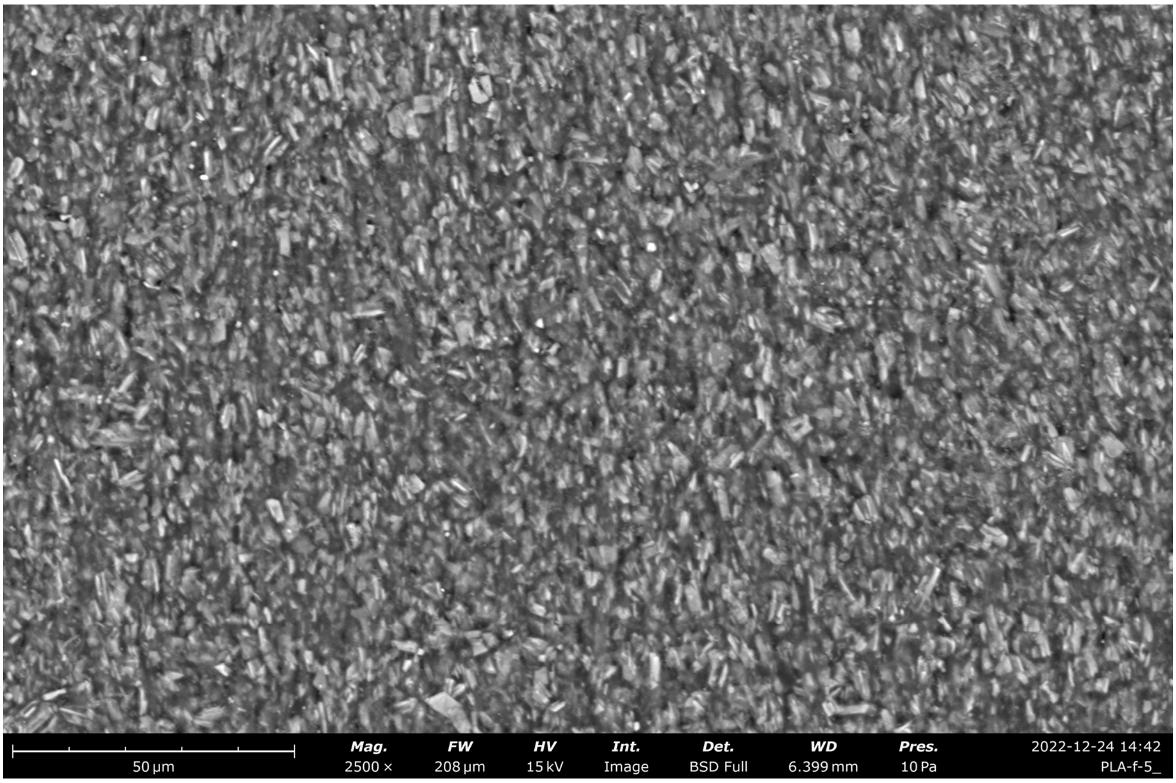

Figure S15. SEM image of the surface of PL13.

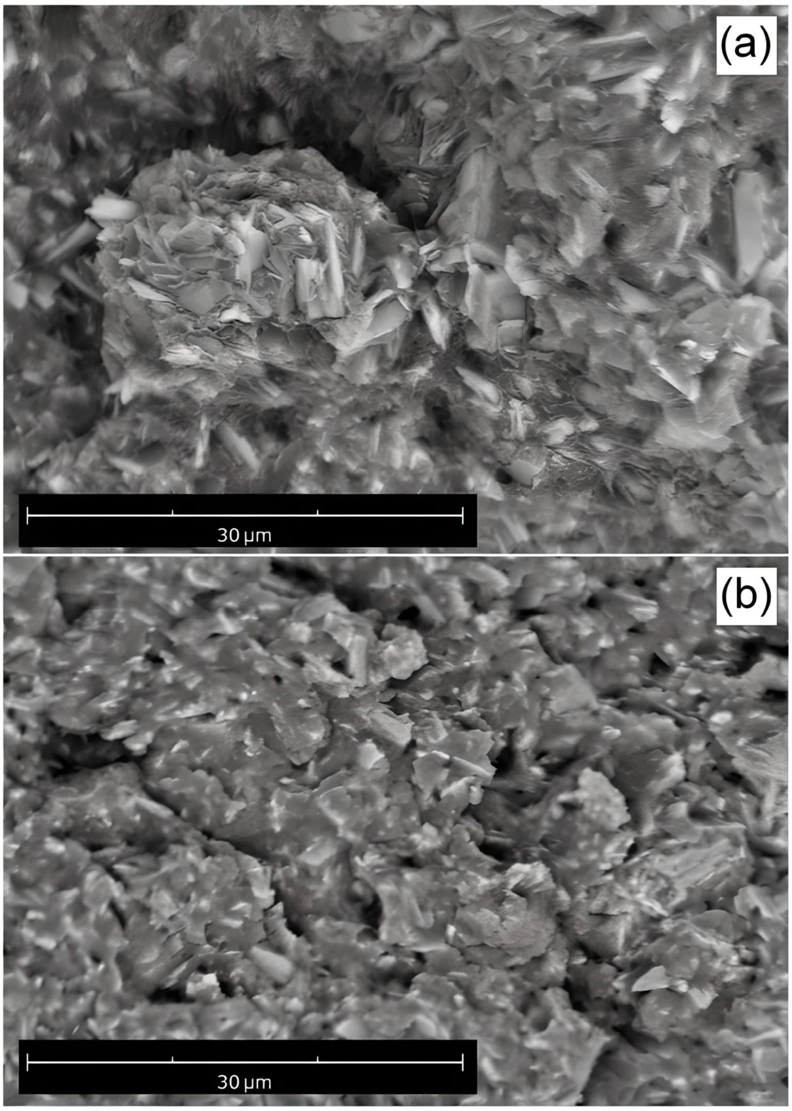

Figure S16. SEM images of the fracture surfaces of samples PL09 (a) and PL13 (b).

S3.3. Contact angle measurements

Contact angle measurements were conducted using plates molded for bending test, for each composite formulation five samples were studied. As can be seen in Table S1, addition of 50 wt% BMS does not lead to substantial decrease of the contact angle. Largest reduction of the contact angle was observed for pCAp-based sample PL13, prepared with the use of C1.

Table S1. Contact angle measurements.

Sample	Contact angle (water, 20 °C)	Photo	Sample	Contact angle (water, 20 °C)	Photo
PLLA	81.2±0.4	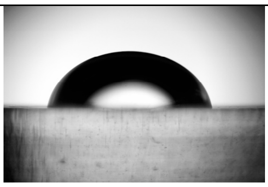			
PL03	73.0±0.6	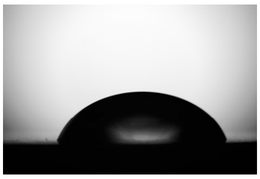	PL09	71.8±0.6	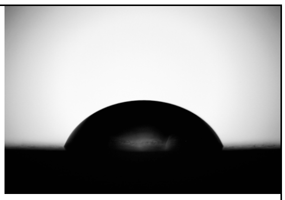
PL06	77.0±0.5	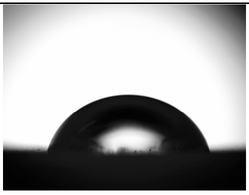	PL13	64.2±0.5	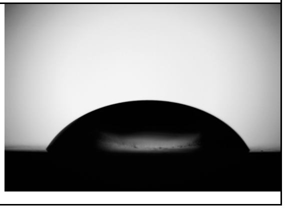

S3.4. Thermal properties of the samples

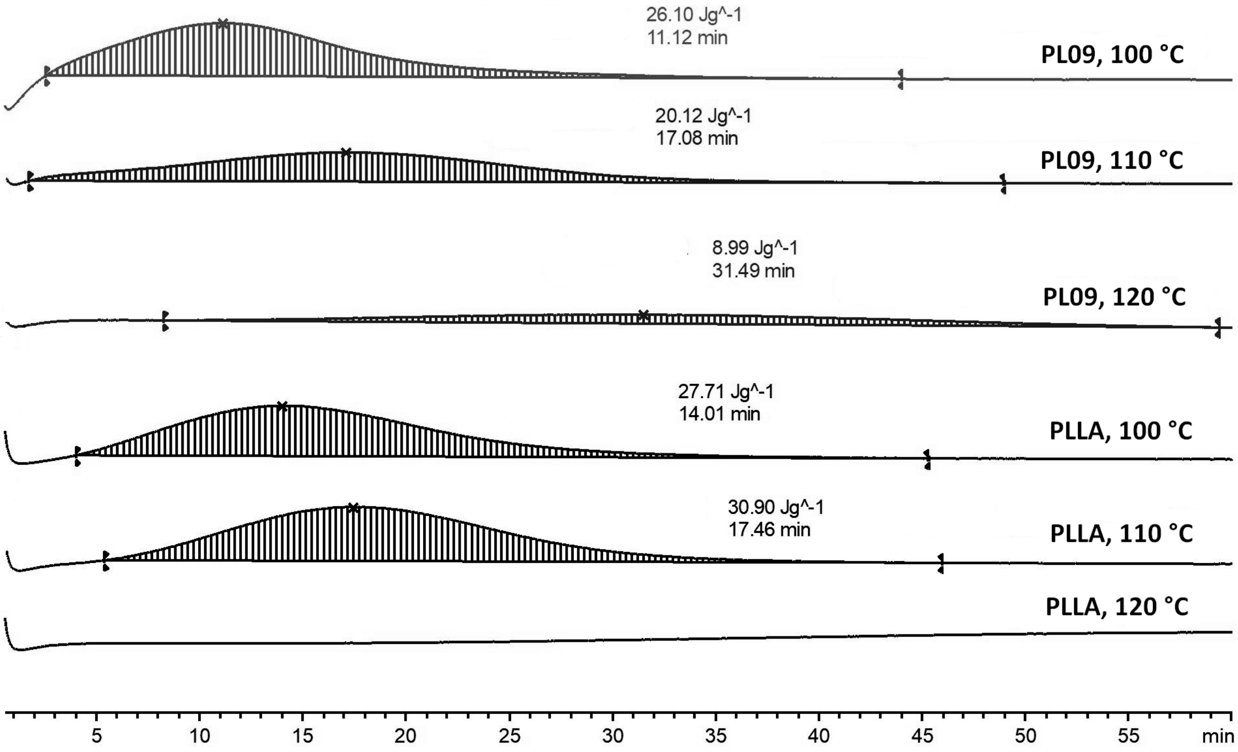

Figure S17. Isothermal crystallization of PLLA and PL09 at 100, 110 and 120 °C.

S3.5. Rheological measurements

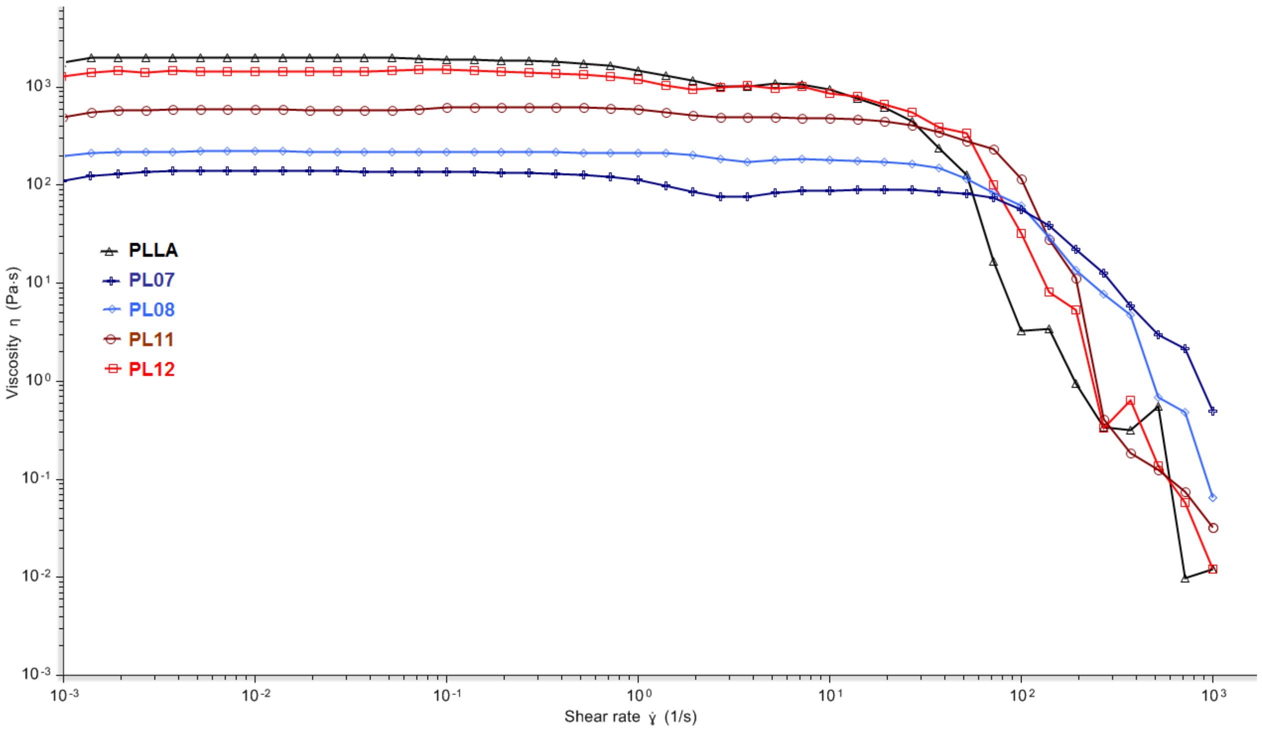

Figure S18. Viscosity (Pa·s) *vs* shear rate (s^{-1}) for PLLA-based composites.

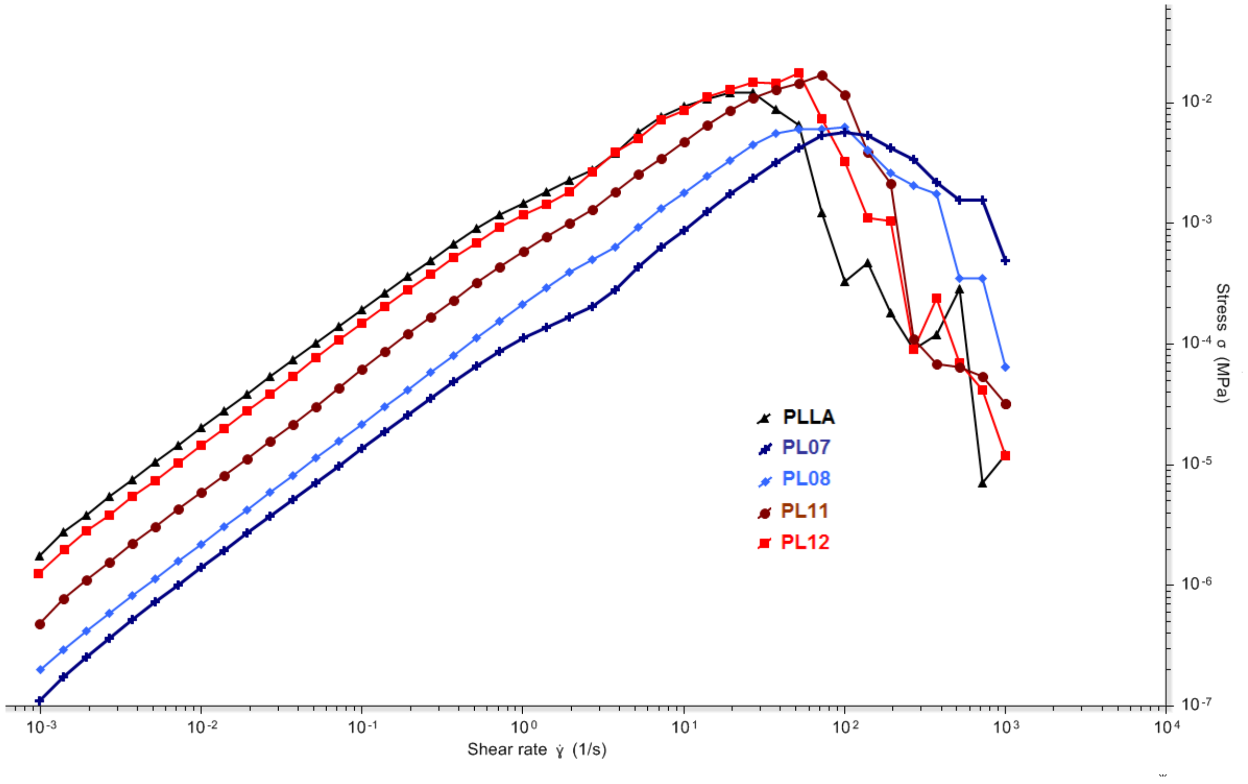

Figure S19. Stress (MPa) *vs* shear rate (s^{-1}) for PLLA-based composites.

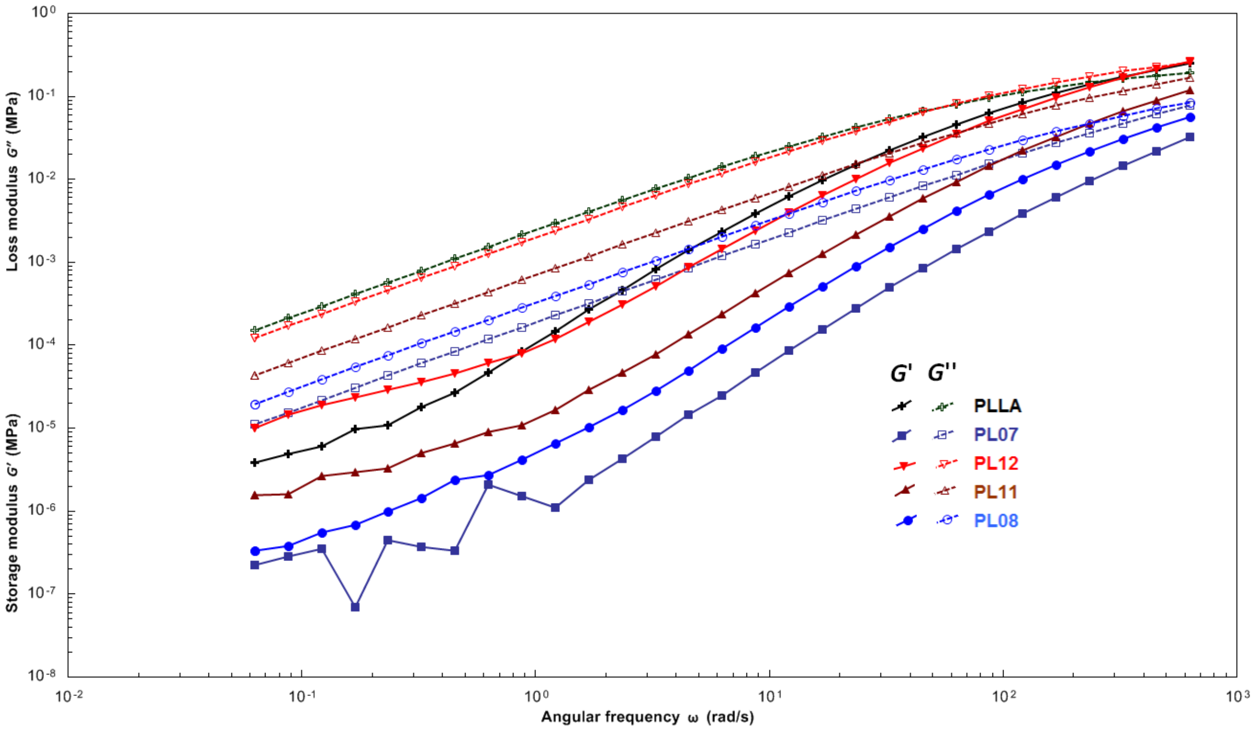

Figure S20. Strorage (G') and loss (G'') moduli *vs* angular frequency (rad·s^{-1}) for PLLA-based composites.

References

1. Nifant'ev, I.E.; Shlyakhtin, A.V.; Bagrov, V.V.; Komarov, P.D.; Kosarev, M.A.; Tavtorkin, A.N.; Minyaev, M.E.; Roznyatovsky, V.A.; Ivchenko, P.V. Controlled ring-opening polymerisation of cyclic phosphates, phosphonates and phosphoramidates catalysed by hereroleptic BHT-alkoxy magnesium complexes. *Polym. Chem.* **2017**, *8*, 6806–6816.
2. Nifant'ev, I.E.; Shlyakhtin, A.V.; Bagrov, V.V.; Komarov, P.D.; Tavtorkin, A.N.; Minyaev, M.E.; Kosarev, M.A.; Ivchenko, P.V. Synthesis in aqueous media of poly(ethylene phosphoric acids) by mild thermolysis of homopolymers and block copolymers based on tert-butyl ethylene phosphate. *Eur. Polym. J.* **2018**, *106*, 249–256.
3. Madupalli, H.; Pavan, B.; Tecklenburg, M.M.J. Carbonate substitution in the mineral component of bone: Discriminating the structural changes, simultaneously imposed by carbonate in A and B sites of apatite. *J. Solid State Chem.* **2017**, *255*, 27–35.
4. Yao, S.; Qi, M.-I.; Qi, L.; Ding, Y.; Chen, M.; Wang, Y. Investigation of EDTA concentration on the size of carbonated flowerlike hydroxyapatite microspheres. *R. Soc. Open Sci.* **2021**, *8*, 202148.
5. Fleet, M.E. Infrared spectra of carbonate apatites: γ^2-Region bands. *Biomaterials* **2009**, *30*, 1473–1481.
6. Grunenwald, A.; Keyser, C.; Sautereau, A.M.; Crubézy, E.; Ludes, B.; Drouet, C. Revisiting carbonate quantification in apatite (bio)minerals: a validated FTIR methodology. *J. Archaeol. Sci.* **2014**, *49*, 134–141.
7. Ren, F.; Ding, Y.; Leng, Y. Infrared spectroscopic characterization of carbonated apatite: A combined experimental and computational study. *J. Biomed. Mater. Res. B Appl. Biomater.* **2014**, *102*, 496–505.
